# Rapid light carbon releases and increased aridity linked to Karoo–Ferrar magmatism during the early Toarcian oceanic anoxic event

**DOI:** 10.1038/s41598-022-08269-y

**Published:** 2022-03-14

**Authors:** Eric Font, Luís Vítor Duarte, Mark J. Dekkers, Celine Remazeilles, Ramon Egli, Jorge E. Spangenberg, Alicia Fantasia, Joana Ribeiro, Elsa Gomes, José Mirão, Thierry Adatte

**Affiliations:** 1 Department of Earth Sciences, University of Coimbra, 3030 790 Coimbra, Portugal; 2 Instituto Dom Luís (IDL), Faculdade de Ciências, Universidade de Lisboa, 1749-026 Lisboa, Lisboa Portugal; 3 MARE and Department of Earth Sciences, University of Coimbra, 3030 790 Coimbra, Portugal; 4 Department of Earth Sciences, Paleomagnetic Laboratory ‘Fort Hoofddijk’, Utrecht University, Princetonlaan 8a, 3584 CB Utrecht, The Netherlands; 5 Laboratoire Des Sciences de L’Ingénieur Pour L’Environnement, UMR CNRS 7356 La Rochelle Université, Pôle Sciences et Technologie, Avenue Michel Crépeau, 17042 La Rochelle Cedex 1, France; 6 Central Institute for Meteorology and Geodynamics (ZAMG), 1190 Wien, Austria; 7 Institute of Earth Surface Dynamics, University of Lausanne, 1015 Lausanne, Switzerland; 8 Univ Lyon, UCBL, ENSL, UJM, CNRS, LGL-TPE, 69622 Villeurbanne, France; 9 Institute of Earth Science - Porto Pole, Rua do campo alegre 687, 4169-007 Porto, Portugal; 10 CITEUC—Centre for Earth and Space Research of the University of Coimbra, 3040-004 Coimbra, Portugal; 11 HERCULES Laboratory, School of Sciences and Technology–Geosciences Department, University of Évora, 7000-671 Évora, Portugal; 12 ISTE, Institut des Sciences de la Terre, Université de Lausanne, GEOPOLIS, 1015 Lausanne, Switzerland

**Keywords:** Climate sciences, Environmental sciences, Solid Earth sciences

## Abstract

Large-scale release of isotopically light carbon is responsible for the carbon isotope excursion (CIE) of the Toarcian Oceanic Anoxic Event during the Lower Jurassic. Proposed sources include methane hydrate dissociation, volcanogenic outgassing of carbon dioxide and/or thermogenic methane release from the Karoo‐Ferrar magmatic province (southern Africa). Distinct small-scale shifts superimposed on the long-term CIE have been interpreted as rapid methane pulses linked to astronomically forced climate changes. In the Peniche reference section (Portugal), these small-scale shifts correspond to distinct brownish marly layers featuring markedly high mercury (Hg) and magnetic mineral concentration. Total organic carbon and Hg increase are uncorrelated, which suggests input of Hg into the atmosphere, possibly released after the intrusion of the Karoo-Ferrar sills into organic-rich sediments. Enhanced magnetic properties are associated with the presence of martite, washed-in oxidized magnetite, inferred to be due to increased aridity on the continental hinterland. This study provides strong evidence for a direct link between the Karoo-Ferrar magmatism, the carbon-isotope shifts and the resulting environmental changes.

## Introduction

**Figure 1:**
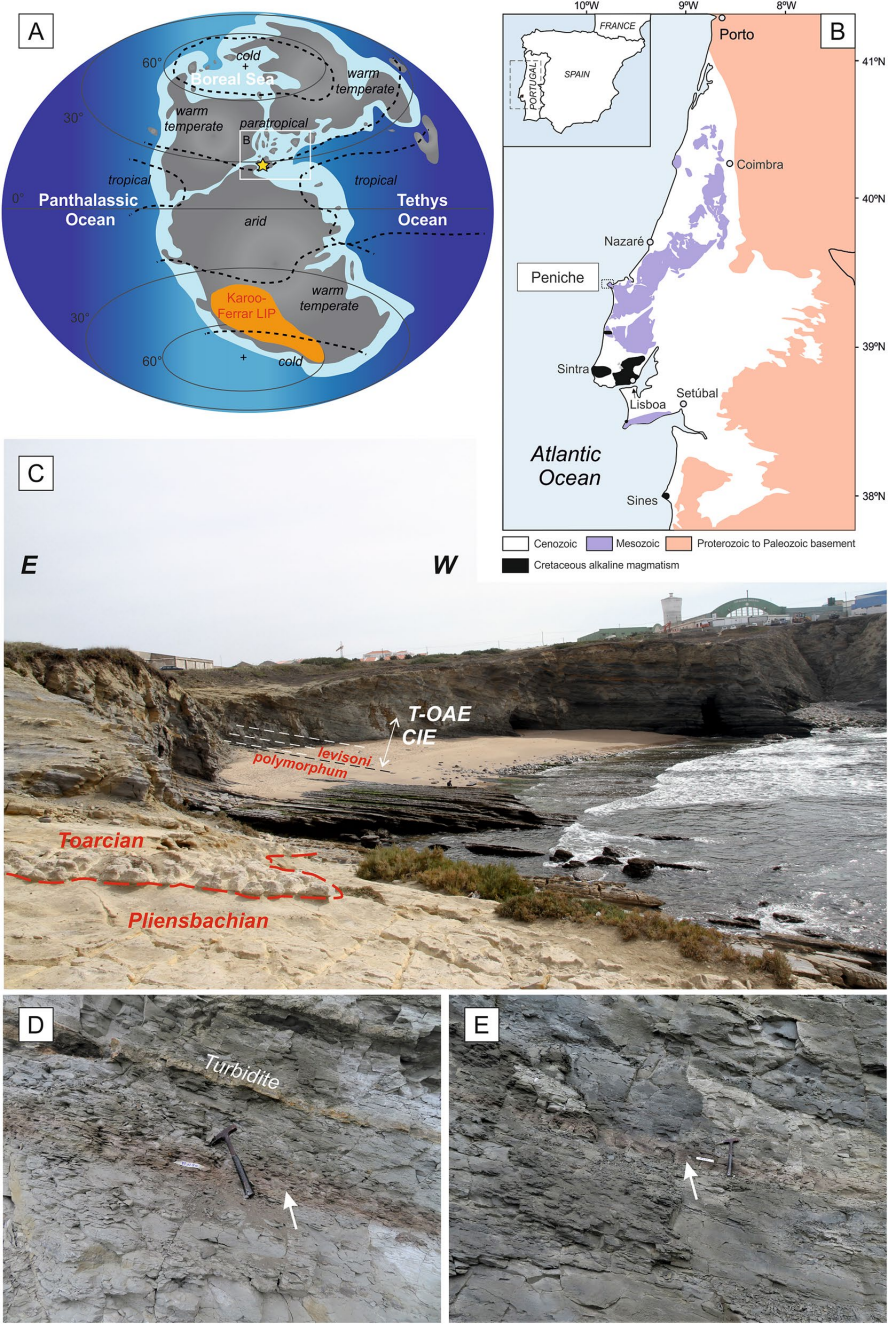
(**A**) Global Toarcian palaeogeographic reconstruction^90^ with the location of the Karoo-Ferrar large igneous province in southern Gondwana, the position of palaeoclimatic belts after^102^, and the location of the Peniche section (yellow star). (**B**) Simplified geological map of the Lusitanian Basin, Portugal. (**C**) Field photographs of the Peniche section. The interval of the Carbon Isotope Excursion (CIE) of the Toarcian Ocean Anoxic Event (T-OAE) is indicated by the white arrow. White dashed lines indicate the position of the three brownish layers. (**D**,**E**) Field photographs of the second (labelled MC2 samples) (**D**) and third (labelled MC3 samples) brownish layers (**E**).

The early Toarcian (early Jurassic) was marked by an episode of global warming (seawater temperature rise of 4–7 °C^1,2^), increase in organic carbon burial, ocean anoxia and mass extinction known as the Toarcian Oceanic Anoxic Event (T-OAE, ~ 183 Ma)^3^, also referred to as the Jenkyns Event e.g.^4,5^. The T-OAE is characterized by one of the largest negative carbon-isotope excursions (CIE; ~ − 2 to − 8‰) of the last 250 Myr^6^ and represents a global perturbation of the carbon cycle e.g.^1,7–11^. This negative CIE has been ascribed to the release of thousands of gigatons of isotopically light carbon into the ocean–atmosphere system^7,8,12–15^. Proposed sources of isotopically light carbon include Karoo-Ferrar magmatism (southern Africa; Fig. 1) and associated release of mantle-derived carbon dioxide^16–21^, thermogenic methane emissions from the intrusion of Karoo-Ferrar sills into Gondwanan coal deposits^13,22^, as well as methane release from marine clathrates and terrestrial environments^7,8,23,24^. The T-OAE carbon-cycle perturbation corresponds to a period of low carbonate productivity and is thought be associated with enhanced hydrological cycling and runoff, resulting in an increased delivery of nutrients and terrestrial organic matter to the ocean^15,25–31^. These processes would have caused an increase in the primary productivity and the development of oxygen-deficient conditions in the oceans leading to an increased burial of organic matter^9,30,32,33^.

**Figure 2:**
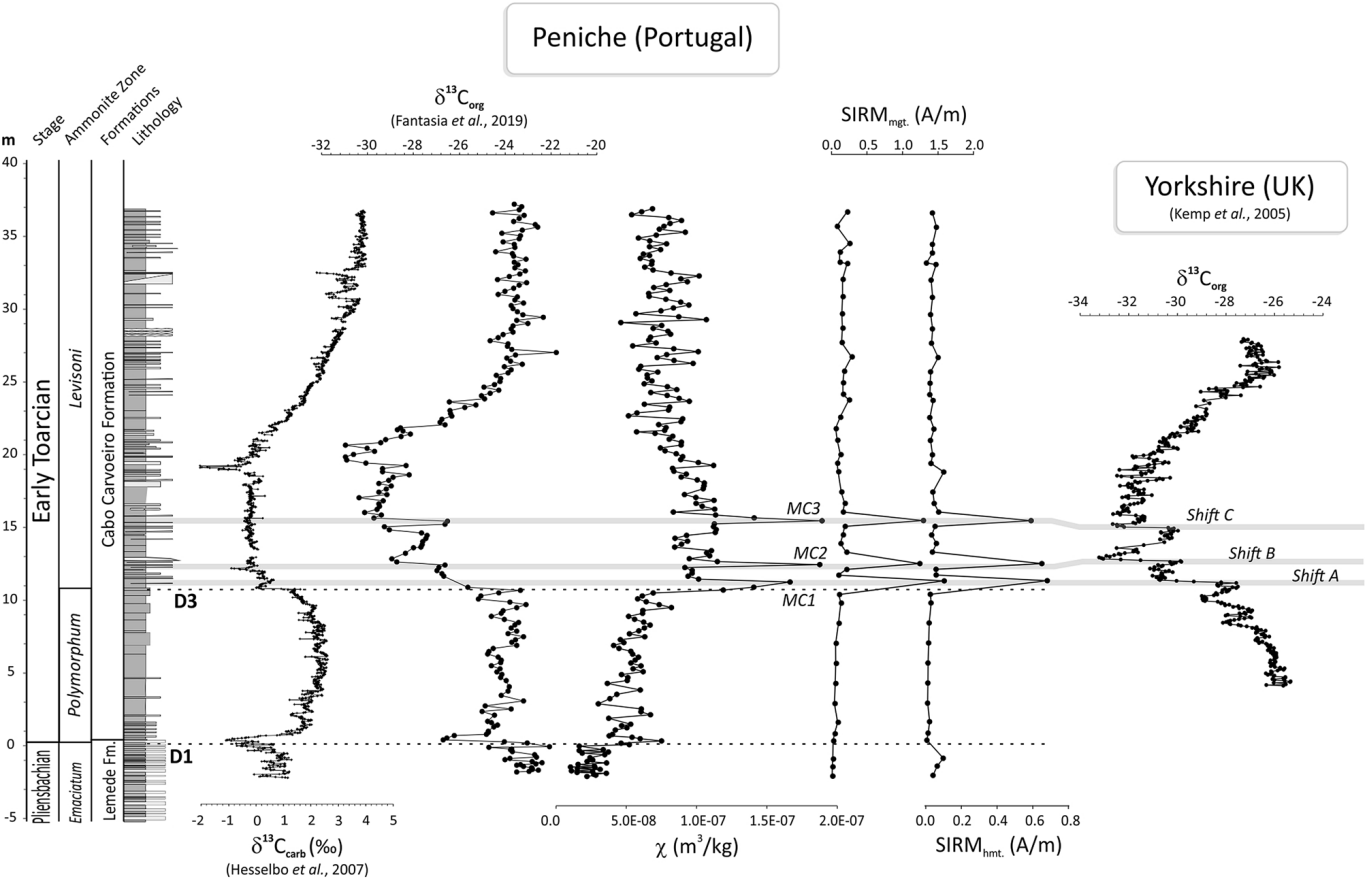
Peniche section: Mass specific magnetic susceptibility (χ) and Isothermal Remanent Magnetization (IRM) parameters after unmixing using the Kruiver et al.^96^ software. SIRM is the saturation of isothermal remanent magnetization. Isotope composition of carbonate (δ^13^C_carb_) and organic carbon (δ^13^C_org_) are from Hesselbo et al.^103^ and Fantasia et al.^31^, respectively. δ^13^C_org_ shifts identified at Yorkshire (UK, Ref.^8^) are shown for comparison. MC1, MC2, and MC3 correspond to the three brownish layers studied here (see also Fig. 3). D1 and D3 correspond to the discontinuities proposed by Pittet et al.^44^.

The long-term CIE (300–500 kyr, Ref.^34–36^) is punctuated by at least three distinct stepwise abrupt δ^13^C negative shifts, with an amplitude of 2–3‰ (vs. VPDB), which are observed in many T-OAE sections, including the Yorkshire section in UK^8^ (Fig. 2), the Sancerre borehole in the Paris Basin^14,24^, France, the Belluno Trough section in Italy^37^, the Mochras Farm section in Wales^37,38^ and the Sakuraguchi-dani section in Japan^29,39^. These distinct shifts have been interpreted as being due to astronomically controlled millennial-scale carbon injections from methane hydrate or terrestrial permafrost sources^8,12,23,29,40,41^. However, the link with magmatic activity from the Karoo-Ferrar province, as well as the climate response to these rapid carbon injections, are unclear. For instance, some authors argue that the Karoo-Ferrar magmatism would have only contributed to the initial decrease of the CIE, whereas the posterior stepwise negative δ^13^C shifts would have resulted from astronomically forced climate change^8,12,40^. To unravel the nature and origin of these short-scale stepwise negative δ^13^C shifts , we provide new high-resolution (cm-scale) records of the organic carbon-isotope composition (δ^13^C_org_), mercury (Hg) content, whole-rock mineralogy, organic matter geochemistry, magnetic properties, and micro-Raman spectra analysis of three stratigraphic levels associated to the distinct δ^13^C shifts of the lowermost portion of the CIE at Peniche (Lusitanian Basin, Portugal), that includes the Toarcian Global boundary Stratotype Section and Point (GSSP)^42^ (Fig. 1).

## Results

Low-field mass specific magnetic susceptibility (χ) of samples from the entire Peniche section (labelled PT samples) varies from 1.14 × 10^–8^ to 1.90 × 10^–7^ m^3^/kg, typical of marine sedimentary rocks (Fig. 2; Table S1 in the supplementary data). Lowest values are observed at the base of the section, in the limestones and marlstones of the topmost Pliensbachian (uppermost part of the Lemede Formation^43^). The Pliensbachian-Toarcian (Pl/To) boundary interval features a rapid shift in χ, which possibly reflects the presence of a condensed interval (the so-called D1 discontinuity described by Pittet et al.^44^). Field observations indicate that the lithology abruptly changes from limestone to marl-dominated sediments. Moving upsection, χ increases gradually along the *polymorphum* Zone and reaches maximum values in the lowermost CIE interval of the *levisoni* Zone (Fig. 2). Further upsection, χ gradually decreases. Particularly noteworthy in the Peniche section are three atypical prominent χ peaks that occur at the same level as rapid shifts in δ^13^C_org_ (Fig. 2) previously determined by Fantasia et al.^31^. These peaks correspond to discrete ~ 5–10 cm-thick distinct brownish marly layers (Fig. 1D,E).

**Figure 3:**
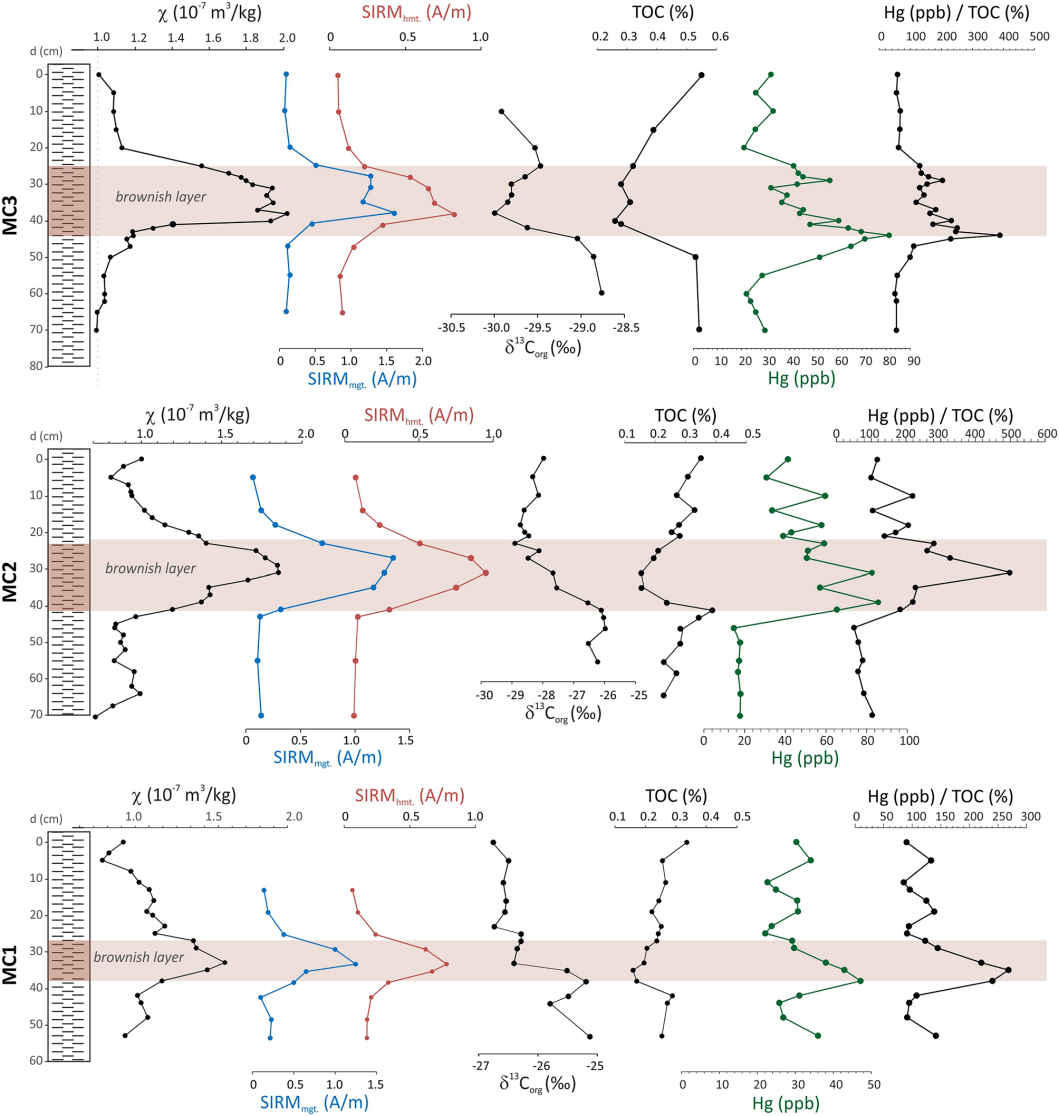
Three brownish layers (MC1, MC2, MC3). From left to right: Magnetic susceptibility (χ), saturation isothermal remanent magnetization (SIRM) of magnetite and hematite after unmixing using the Maxunmix software^97^, organic carbon isotope composition (δ^13^C_org_), total organic carbon (TOC), mercury content and Hg/TOC ratio. These distinct layers are coeval to the three rapid shifts in carbon isotope composition (shifts **A**–**C**) illustrated in Fig. 2.

Two principal ferromagnetic phases are identified in the early Toarcian of the Peniche sediments based on the unmixing of Isothermal Remanent Magnetization (IRM) acquisition curves (Fig. S1 in the supplementary data). The low-coercivity phase is characterized by a skewed coercivity distribution modelled by two Gaussian functions (components 1 and 2) with median acquisition fields (noted as B_1/2_) of ~ 15–25 mT and ~ 45–55 mT and dispersion parameter (DP) of 0.2–0.4 log mT and 0.27–0.34 log mT, respectively. First-order reversal curves (FORC) (Fig. S2) show that this low-coercivity phase is composed by ~ 97% of > 0.1 µm detrital (titano-)magnetite with the typical signature found in sedimentary rocks, and for the remaining ~ 3% by < 0.1 µm magnetite, possibly of secondary origin^45–48^. The lack of biogenic (secondary) magnetite, typically identified by DP < 0.2 and a narrow central ridge in FORC diagrams, and which is generally abundant in pelagic sediments^49^, is common in hemipelagic environments with high sedimentation rate and low TOC (Peniche: ~ 1 m/kyr during the early Toarcian cf.^50^). The ratio of components 1 and 2 is relatively constant at ~ 1:19 throughout the section (Fig. S3), confirming their common origin from a single mineral phase with fixed properties. The high-coercivity phase is represented by a single Gaussian function (component 3) with B_1/2_ of ~ 200–400 mT and DP of 0.30–0.50 (log mT), typical of hematite. The relative content in magnetite (components 1 and 2) and hematite (component 3) is expressed by the values of the IRM at saturation (SIRM) of the corresponding components in Figs. 2 and 3. SIRM of magnetite and hematite is low in most samples but typically one order of magnitude higher in the brownish levels. In these levels hematite’s contribution to the SIRM increases from a base-line value of ~ 15 to ~ 37%.

To uncover the environmental significance of these magnetically enhanced brownish levels associated with the rapid δ^13^C shifts, we measured their χ, IRM, δ^13^C_org_, TOC and bulk Hg content at a cm-scale resolution (MC transects; Fig. 3). Both the base and top of the brownish layers feature low χ and SIRM values with a gradual increase towards maximum values more to their centers (Fig. 3). Brownish layers show B_1/2_ values of 350–400 mT for the high-coercivity component 3 while surrounding sediments have lower values of ~ 200–280 mT (Fig. S3 and Table S3 in the supplementary data), suggesting that the former contain a distinct population of hematite. δ^13^C_org_ shifts of 2–3‰ previously obtained by^31^ in Peniche and by^8^ in Yorkshire are confirmed here by our new measurements illustrated in Fig. 2. These shifts, named A, B and C in Yorkshire^8^ (Fig. 2), are shown to be abrupt in the case of the first (MC1) and third (MC3) brownish layer at Peniche, occurring within a 5–7 cm-thick interval. In these levels concentration-dependent magnetic properties (χ and SIRM) change abruptly as well (Fig. 3; Table S4 in the supplementary data). The second brown layer (MC2) shows more gradual shifts in measured parameters within a 14 cm-thick interval. The three brownish layers also feature lower TOC contents of ~ 0.1–0.2% compared to the over- and underlying sediments which have twice these values (~ 0.2–0.6%, Table S4). Notably, Hg varies from 20–30 ppb in the over- and underlying sediments up to 50–85 ppb in the brownish layers. This Hg enrichment is coeval with the increase in χ and SIRM and the decrease in δ^13^C_org_ observed in these brown beds (Fig. 3, Table S4).

**Figure 4:**
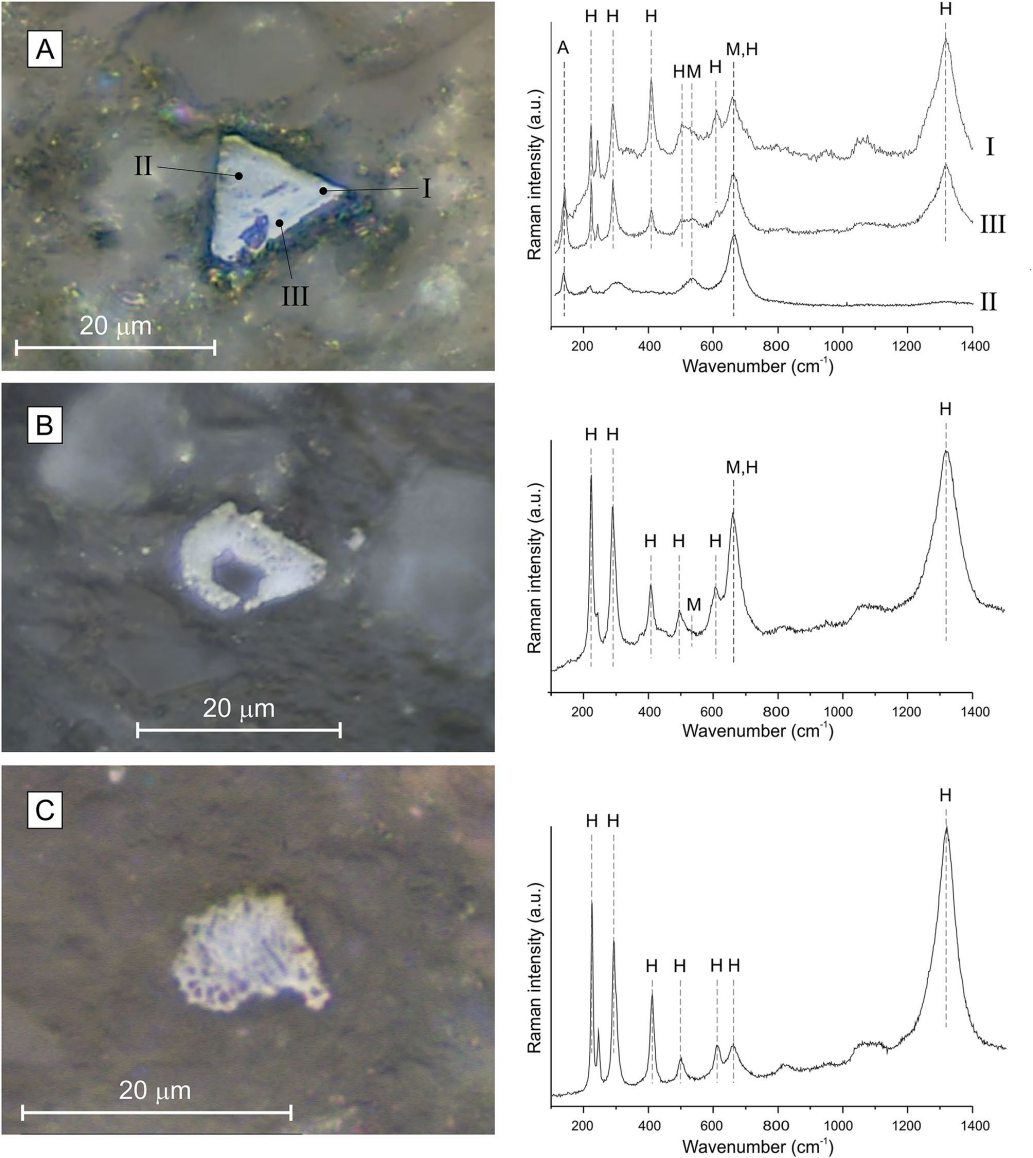
Typical reflected light microscopic photographs of martite and corresponding micro-Raman spectra observed in the brownish layers. Particles show the typical habit of magnetite with a mixed composition of magnetite (M) and hematite (H). I, II and II in the top right panel belong to spots I to III.

Optical microscopy coupled to micro-Raman analyses of six MC samples revealed the presence of non-magnetic Ti-oxides (mainly anatase and rutile) in all samples (Fig. S5 in the supplementary data), possibly of a detrital origin or as the product of reductive dissolution of primary iron oxides in a sulfidic anoxic environment. All samples contain abundant framboidal pyrite, mainly associated to organic matter (Fig. S6). Pyrite framboids of heterogeneous grainsize associated with wood fragments suggest that they did not nucleate in the water column but formed after sedimentation, by alteration of organic matter. Well-preserved pyrite and the overall absence of alteration to hematite or goethite in the Raman spectra indicate that the samples did not suffer late diagenetic alteration or oxidation (Fig. S5-S6). The brownish layers contain abundant Ti-bearing iron oxides (Fig. S7), which are exceedingly rare or completely absent in other samples. Crystals exhibit a cubic to octahedral habit typical of magnetite (Fig. S7.A-E) with different degree of alteration expressed by dissolution and exsolution features (Fig. S7). Semi-hexagonal habits typical of hematite or ilmenite are also observed (Fig. S7.G-H). Micro-Raman analyses reveal that some crystals are made of a mixed composition of magnetite and hematite, whereas others are entirely hematite, suggesting that the magnetite host underwent significant martitization (Fig. 4). Relics of magnetite in hematite are rare but were identified under reflected light microscopy (plane and crossed polarized light) in sample MC3-33 (Fig. S7.I). Some hematite crystals have subhedral forms (Fig. S7.J), typical of magnetite, but, occasionally, tabular hematite crystals are observed (Fig. S7.K).

## Discussion

A significant magmatic emplacement event has been linked to major changes in the T-OAE paleoenvironment. Specifically, the timing of the emplacement of the Karoo-Ferrar large igneous province (LIP) appears to coincide with the Pl/To mass extinction, recorded in marine and terrestrial sedimentary settings^16,17,51^. Paleomagnetism and geochronology (U–Pb and Ar-dating) of intrusive and extrusive magmatic rocks from the Karoo and Ferrar LIPs bracket the main emplacement event to 182–183 Ma, with a duration of 300–500 kyr^19,20,52,53^. The age of the Pl/To boundary is placed at 183.6 + 1.7/− 1.1 Ma based on U–Pb geochronology and biostratigraphy^17^. Based on the correlation of U–Pb dates of zircon from ash beds deposited during the early Toarcian in Palquilla (southern Peru) with previously published ages of Karoo intrusives, Sell et al.^54^ place the Pl/To boundary at no younger than 183.5 Ma. Starting from an age of 183.5 Ma for the Pl/To boundary, Sell et al.^54^ and Burgess et al.^55^ argue that Ferrar magmatism begins within uncertainty at the onset of the T-OAE and the CIE, and continues through this interval. Based on high-precision U–Pb and ^40^Ar/^39^Ar geochronology, Greber et al.^56^ suggest that Karoo-LIP magmatism occurred after the Pl/To boundary and therefore postdated the extinction, but support the onset of Karoo-LIP activity and the T-OAE and the early Toarcian warming being synchronous. However, the resolution of radiometric methods does not allow for a more precise correlation between the discrete stepwise negative δ^13^C shifts within the CIE and their possible linkage to the emplacement and eruptive history of the Karoo-Ferrar LIP. Also, the age estimate of the Pl/To boundary is not well-constrained enough to definitively link it to the Karoo-Ferrar LIP.

The stepwise pattern of the negative CIE and presence of distinct δ^13^C shifts have been tentatively linked to Karoo-Ferrar LIP magmatism following two model scenarios. The first scenario invokes the liberation of huge amounts of isotopically light carbon to the ocean–atmosphere system through methane hydrate dissociation controlled by astronomically forced climate variations^8,12,40^. In this model scenario, volcanogenic CO_2_ from the Karoo-Ferrar magmatic events would not have effused fast enough to cause the δ^13^C shifts. Instead, magmatism would have only contributed to the slower initial ~ 2‰ decrease in δ^13^C that began before the first methane hydrate dissociation pulse inferred from the Yorkshire record^8^ (Fig. 2). Global warming from volcanic outgassing might then have acted in concert with astronomical climate forcing to drive episodic dissociation of methane hydrate, resulting in distinct stepped negative δ^13^C shifts. This hypothesis is supported by spectral analysis of carbon-isotope and carbonate abundance that revealed a regular cyclicity, compatible with precession^8,40^ or obliquity^12^. Assuming that the astronomical forcing is controlled by orbital precession cycles (~ 21 kyr in the early Jurassic), Kemp et al.^8^ estimated the duration of the three pulses of methane hydrate dissociation to < 2 kyr each.

The second scenario implies a direct link between the Karoo-Ferrar eruptions and the stepped δ^13^C shifts, either by repeated pulses of massive degassing of volcanogenic CO_2_^1,15^ or by the release of thermogenic CO_2_ and CH_4_ from organic-rich sediments in southern Gondwana induced by the intrusion of magma^13,22^. These magmatic processes are not predicted to exhibit astronomical periodicity that would explain the cyclic nature of the stepwise δ^13^C shifts. Both hypotheses can be tested with high-resolution Hg chemostratigraphy. Hg has been used to infer the causal relationship between LIP activity and extinction events recorded in marine and terrestrial sedimentary rocks e.g.,^57–62^. Hg enrichments recorded during the T-OAE interval at several sites from Europe and the southern hemisphere have been interpreted as evidence for a causal link between Karoo-Ferrar volcanism and coeval environmental changes^31,62^. In the Peniche section abrupt and short-lived positive excursions in Hg concentrations and Hg/TOC ratios coincide with the Pl/To boundary and with the onset of the negative CIE during the T-OAE^62^. However, a precise correlation of Hg content and the distinct δ^13^C shifts is beyond the sample resolution of those studies (10–50 cm in^62^; 20 cm in^31^). The resolution in our data set (2–5 cm sample spacing) allows for these two scenarios to be investigated. Findings suggest a direct link between the distinct stepwise pattern that is superposed on the negative CIE and magmatic pulses associated with Karoo-Ferrar activity. We show that the distinct discrete brownish marly layers correspond to the stratigraphic intervals where rapid shifts in δ^13^C_org_ (called Shift A, B and C by analogy with the Yorkshire section, Fig. 2) were documented previously^31^. An absence of significant variations in oxygen (OI) and hydrogen (HI) values (Table S4) suggests that the decrease in δ^13^C_org_ values cannot be explained by a change in the nature and composition of the organic matter (see also^63^). Nonetheless, each shift is associated with increased Hg content (Fig. 3). For example, shift B shows a rapid and striking increase up to 85 ppb Hg, coeval with the onset of the δ^13^C_org_ decrease. Hg contents then gradually decrease concomitantly with the decrease in δ^13^C_org_ values (Fig. 3) suggesting that these events are linked. Due to the fact that Hg has a strong affinity for organic matter, correlated Hg and TOC values in modern and ancient sediments are generally taken to reflect the input of Hg bound to organic matter or detrital particles (clays) from terrestrial run-off^57,60,64^. Alternatively, some authors^64^ infer that Hg enrichment within the T-OAE interval may be caused by local redox conditions and/or remobilization of terrestrial Hg. At Peniche, the general increase in Hg background values suggest a link between the T-OAE and the onset of Karoo-Ferrar volcanic activity^31,62^. We noted that localized factors, such as the presence of pyritized wood fragments could also have affected the Hg record^31^. However, in the case of the brown layers, Hg does not correlate with TOC (Fig. 3) or clay contents (Fig. S7 in the supplementary data). In the Hg enriched brownish layers, TOC are at least two times lower that in the over- and underlying sediments (Fig. 3). Because pyrite is mainly associated with organic matter (Fig. S4 in the supplementary data), lower TOC would also point to lower pyrite contents, suggesting that post-depositional Hg remobilization and fixation by pyrite is not the cause of the Hg enrichment. After normalizing for varying TOC levels, Hg/TOC peaks remain a prominent feature within the brownish marls, with values five times higher than background levels (Fig. 3). Note that Grasby et al.^57^ suggest that very low TOC contents close to the limit of detection may lead to spurious results and misinterpretation and proposed that Hg/TOC values should not be calculated for < 0.2wt% TOC. For this reason, we only consider the bulk, non-normalized, values of Hg. With or without normalization to TOC, these data suggest that a rapid Hg increase is coeval with a rapid δ^13^C_org_ shift, which we infer to be from direct atmospheric deposition of Hg sourced from Karoo-Ferrar magmatic events. Whether this Hg increase results directly from volcanic outgassing or corresponds to liberation of thermogenic Hg after the intrusion of Karoo-Ferrar magma into organic-rich sediments remains to be elucidated.

As volcanogenic carbon has δ^13^C < -6‰, previous workers suggested that the Karoo-Ferrar volcanic eruptions would have not effused fast enough to directly cause the largest T-OAE excursion recorded worldwide^7,8,65^. Volcanic degassing is a potentially large but poorly constrained source of mercury^66^. Basaltic rocks with minimal crustal contamination have generally low Hg concentrations ranging from 0.2 to 7 ppb^67–69^. For example, the Karoo dolerite sill (chilled margins) have average Hg concentrations of 2.5 ppb^67^. The low concentration of Hg in mantle-derived magmatic rocks is due to the fact that Hg is a chalcophile element and behaves as an incompatible element with silicate mineral assemblages. The amount of gaseous elemental mercury released during the eruptions of continental flood basalts will thus mainly depend on the Hg contamination from the crust. Most of the Karoo-Ferrar LIP is represented by sills and dykes intruded into Carboniferous to early Jurassic organic-rich sediments^22^. Based on mass balance calculations constrained by contact aureole around the sills, Svensen et al.^22^ suggested that 27,400 Gt of CO_2_ may have been released from the entire Karoo basin during the intrusive event. The released gas would have a δ^13^C isotopic composition close to that of bulk oil and organic matter (~ − 30 to − 25‰^70^); at the same time significant amounts of Hg are released from the organic-rich sediments. Decomposition of gas hydrate deposits associated to hydrothermal activity, such as in the eastern shelf of Sakhalin (Russia), may release significant amounts of mercury into shelf waters^71^. In this case, Hg is supplied by hydrothermal fluids from deeper fault zones. However, no evidence of hydrothermalism is observed at Peniche. In addition, the main argument against the hypothesis that methane hydrate destabilization was the driver for the three discrete δ^13^C shifts discussed here, relies on the time needed to recharge the methane hydrate reservoir. The duration of the entire Toarcian CIE has been estimated to be comprised between 300 and 900 kyr^36,72^. A more conservative estimate of 300–500 kyr has been calculated for Peniche, Sancerre (Paris), Yorkshire (UK) and the Talghemt section (High Atlas in Morocco)^34,35^. With a thickness of ~ 10 m for the negative CIE at Peniche (Fig. 2), a sedimentation rate of ~ 2 to ~ 3.3 cm/kyr would result. Considering that each brownish layer is spaced by 1–3 m, the time span between each δ^13^C shift is ~ 50 to ~ 150 kyr. These durations are way too short for the methane hydrate reservoir to be recharged as explained next. Based on coupled climate model simulations of orbitally induced changes in ocean circulation and intermediate water temperature during the Paleocene-Eocene Thermal Maximum, Lunt et al.^73^ calculated that the submarine gas hydrate stores recharge on a timescale of 500 kyr, i.e. 3 to 10 times longer than the interval at Peniche. Using the GEOCLIM carbon cycle model, Heimdal et al.^74^ show that a total of 20,500 Gt C replicates the Toarcian pCO_2_ and δ^13^C proxy data, and that thermogenic carbon represents a plausible source for the observed negative CIEs. In this scenario, the extremely isotopically depleted carbon signature of methane clathrates is not required to replicate the negative CIEs. Therefore, we suggest that the Hg enrichment associated with the distinctively stepwise abrupt δ^13^C negative shifts observed at Peniche is better explained by the rapid, sporadic and massive release of thermogenic carbon and Hg from intrusive events of the Karoo-Ferrar LIP.

Interestingly, the three distinctive brownish beds straddling the δ^13^C_org_ shifts are characterized by higher Hg levels and show a strong increase in magnetite and hematite concentration (Figs. 2 and 3). Micro-Raman analyses and optical microscopy reveal that magnetite and hematite correspond to martites (Fig. 4), hematite pseudomorphic after magnetite that typically forms under oxidizing conditions (e.g.,^75^). Provenance changes (composition of the sediment source, aeolian contribution, etc.) and sea-level changes may result in fluctuations of the magnetic signal in marine sediments. This pertains to the relative contribution of diamagnetic calcite from carbonate productivity and dia-/para-/ferromagnetic materials washed-in from terrestrial detrital sources. Also, diagenesis and precipitation of new magnetic minerals at the redox front of the sediment/water interface may yield magnetic anomalies^76^. This goes as well for aeolian input of dusts rich in magnetic particles or climate induced production of magnetic soils, both of which result in a magnetically enhanced detrital input. In the Peniche section, phyllosilicate and calcite are uncorrelated to χ in the brown bed which indicates that the enhanced magnetization is not primarily controlled by lithology in these layers, or by sea-level fluctuations (Fig. S8). In addition, the stratigraphic positions of the brownish layers do not correspond to stratigraphic discontinuities, like those described by Pittet et al.^44^ at the Pl/To boundary (D1) and at the *polymorphum-levisoni* zonal transition (D3), that likely reflect transgression-related sediment starvation (Fig. 2). The close association of the magnetically enhanced levels with the distinct δ^13^C shifts while the remainder of the section features background magnetic levels, together with the presence of well-preserved pyrite also argue against a late diagenetic imprint due to burial, fluid circulation or oxidation. Rather, the close relationship between the sediment color, increased Hg contents and δ^13^C_org_ shifts suggests that magnetic enhancement and the presence of martite are the consequence of climate and environmental changes triggered by magmatic activity of the Karoo-Ferrar LIP. Martitization is a widespread phenomenon in various geological settings, however the processes involved in its formation are not fully understood (e.g.,^75,77^). Martite occurs in banded iron formations, ore deposits and continental red beds but has been rarely described in marine hemipelagic sediments such as those studied here. The transformation of magnetite into hematite has been considered a redox reaction linked to increased oxygen fugacity. However, the oxygen fugacity in many geologic environments is deemed too low to account for hematite formation via classic redox reactions, leading some authors to consider non-redox formation reactions involving the leaching of Fe^2+^ ions by acidic solutions^75,77,78^. Although oxygen fugacity is relatively low in hemipelagic and pelagic settings, redox transformation of magnetic minerals may occur in some organic-rich marine sediments. It has, for example, been documented in so called “burnt down” fronts of Mediterranean sapropels^76,79–83^. Sapropels are deposited under anoxic conditions, and they can be partially or completely oxidized upon re-establishment of oxygenated bottom water ventilation^84,85^. During this oxidation, authigenic iron oxides are precipitated, which leads to enhanced magnetizations in the oxidation front. Upon re-establishing the oxygen diffusion into the sediment, it reacts with Fe^2+^ liberated from pyrite and also with upward-diffusing Fe^2+^ from the organic rich sapropel sediments below the oxidation front. However, in contrast to the present study, magnetite is the main magnetic mineral formed in the sapropel oxidation front: hematite is barely or not at all formed^79–82^. When present, the minute hematite contribution is thought to reflect variations in the input of aeolian (Saharan) dust^86^. The entire Peniche section is characterized by low concentrations of redox sensitive elements (V and Mo) indicating that the depositional conditions were not favorable to the development of long-term anoxic conditions, but was rather dysoxic^31^. Pyrite framboids are formed in situ and precipitated in close association with organic matter (Fig. S6). They do not show any signs of oxidation or dissolution under scanning electron and optical microscopes, or compositional changes in the Raman spectra (Fig. S6). Periods of intermittent bottom-water oxygenation can be created by turbidites, which are rather frequent in the Peniche section during the *levisoni* Zone^87,88^. However, no turbidites are associated with the brownish layers; they are documented at least a few decimeters away from them (Fig. 1D,E). In-situ martitization by non-redox reactions involving acid solutions is also unlikely given the buffering capacity of the prevalent carbonates in the sediment. Therefore, we argue that martitization took place in the hinterland, followed by martite deposition in the sedimentary basin. For instance, martite has been found in the C-horizon of loessic soils, where it forms as weathering product of parent minerals^89^. The increased relative concentration of the hematite magnetic component in the brown levels can thus be ascribed to episodes of increased soil erosion. We note here that Peniche is located at the boundary between warm temperate and arid climate during the Toarcian^90^ (Fig. 1A). Oxic and arid conditions on the continent during the *levisoni* Zone were also suggested by Rodrigues et al.^91^ based on the characterization of the terrestrial organic matter. The region is thus sensitive to record rapid and brief shifts in paleoclimatic belts, which provides an explanation for the distinctive magnetic signature of the brownish layers. Increased aridity would have resulted in enhanced martitization, as well as in a higher level of mineralization of the organic matter and/or lower productivity, explaining the lower TOC values observed in these samples. The brownish layers also appear to contain the lowest amounts of calcite (Fig. S8), which indicates reduced carbonate production. Lower carbonate production during the T-OAE is hypothesized to have been at least partly triggered by ocean acidification linked to increased atmospheric pCO_2_^92,93^. The boron isotopic composition (δ^11^B) of brachiopod shells from Peniche shows that the seawater pH decreased ~ 0.6 units just prior to and within the T-OAE, in conjunction with a lower CaCO_3_ content in the sediments, concurring with changes in atmospheric pCO_2_ derived from stomatal indices^94,95^. In this context, martitization on the continent could have been possibly mediated by more acid conditions due to a higher pCO_2_ and SO_2_ release triggered by Karoo-Ferrar magmatic activity.

## Conclusions

We show that stepwise abrupt δ^13^C negative shifts observed in many T-OAE section worldwide and featuring the onset of the long-term early Toarcian carbon isotope excursion, are represented at Peniche by discrete (~ 5–10 cm-thick) layers with a marked brownish colour, and distinct geochemical and mineralogical properties. These brownish layers exhibit increased Hg contents and Hg/TOC ratios concomitant with the rapid decrease of δ^13^C of ~ 2–3‰, suggesting a direct link between the Karoo-Ferrar magmatism and the rapid injection of light carbon into the atmosphere. The time span between the deposition of each brownish layer is too short for the discrete shift to be due to destabilization of methane clathrates but can, instead, be explained by sporadic and massive release of thermogenic carbon dioxide and methane, together with Hg, from the intrusion of Karoo-Ferrar sills into organic-rich sedimentary host rocks. In addition, we show that the increased magnetic properties of these brownish layers result from the presence of martite, i.e., hematite pseudomorphs after magnetite that typically form under conditions of increased oxygen fugacity. The presence of these martites is an indicator of palaeoenvironmental and palaeoclimate change, specifically increased aridity, caused by the early Toarcian global warming event.

## Methods

### Study locations

The Pliensbachian and Toarcian of the Peniche section crops out on the western coast of Portugal and is generally composed of an alternation of hemipelagic marl and limestone deposited on a homoclinal ramp system in the distal part of the Lusitanian Basin, e.g.,^43^ (Fig. 1). During the early Toarcian, the Peniche area received siliciclastic sediments from the emerged Berlenga-Farilhões Horst located to the west^87,88^. The studied interval is about 35 m thick and includes the *emaciatum* ammonite-Zone of the upper Pliensbachian, and the *polymorphum* and *levisoni* Zones of the lower Toarcian^43^ (Fig. 2). The upper ammonite Zone belongs to the Lemede Formation (Pliensbachian) and consists of decimetric-thick alternations of bioturbated limestone and marlstone. The beds often show shell accumulations and are capped by a condensed interval just above the Pl/To boundary (topmost portion of the Lemede Formation). The lower Toarcian (Cabo Carvoeiro Formation) is locally characterized by bioturbated marl-dominated deposits. The lower-middle part of the *levisoni* Zone contains mixed-carbonate siliciclastic turbidites and abundant wood fragments. Detailed mineralogical and geochemical data of this interval of the Peniche section, including clay mineralogy, organic matter geochemistry, major and trace elements, and carbon isotope stratigraphy are published in Fantasia et al.^31^.

### Sample collection and processing

Magnetic susceptibility (χ) and Isothermal Remanent Magnetization (IRM) data shown in Fig. 2 were measured on 210 rock fragments (labelled PT) collected with a sampling spacing of ~ 20 cm along the entire ~ 35 m-thick of the Peniche section. These samples correspond to the same samples previously studied by Fantasia et al.^31^. Samples from the three levels of high magnetic susceptibility (labelled MC1 with 20 samples, MC2 with 33 samples and MC3 with 28 samples) corresponding to the brownish layers associated with the rapid δ^13^C_org_ shifts A, B and C (Fig. 3) were collected at a later stage with a higher, cm-scale, sampling resolution. The MC samples were crushed in the laboratory using an agate mortar.

Magnetic measurements were performed at the Paleomagnetism Laboratory of the Instituto Dom Luís (IDL) of the University of Lisbon and the Department of Earth Sciences of the University of Coimbra, both in Portugal. Magnetic properties of the MC samples include the measurement of mass specific magnetic susceptibility (χ, in m^3^/kg) and determination of IRM acquisition curves. Stepwise IRM (typically 30 steps) was acquired with an impulse magnetizer (model IM-30; ASC Scientific) and measured with a JR6 magnetometer (AGICO; measurement range up to 12,500 A/m; noise level and sensitivity of 2.4 × 10^−6^ A/m for a standard-sized (10 cc) paleomagnetic sample). The maximum applied field was 1.2 T. IRM acquisition curves were unmixed into several components based on cumulative log-Gaussian (CLG) functions with the Kruiver et al.^96^ software or on skewed generalized (log) Gaussian functions with the Max UnMix program (Maxbauer et al.^97^) to isolate the contributions of magnetite and hematite (denoted here as SIRM of component 1–2 and component 3, respectively). One sample from MC3 was further characterized with high-resolution FORC measurements performed with a Lake Shore 8600 Vibrating Sample Magnetometer at the material magnetism laboratory of the Central Institute of Meteorology and Geodynamics in Austria (13 stacks of 851 curves each, acquired in regular field steps of 0.35 mT) and processed with VARIFORC^98,99^.

Carbon isotope compositions of bulk organic matter (δ^13^C_org_, ‰ VPDB) were determined on decarbonated (10% HCl treatment) powdered whole-rock samples by flash combustion, using an elemental analyser Carlo Erba 1108 elemental analyser connected to a Thermo Fisher Scientific Delta V Plus isotope ratio mass spectrometer at the Institute of Earth Surface Dynamics of the University of Lausanne (IDYST–UNIL), Switzerland. The repeatability and intermediate precision of the δ^13^C_org_ values was better than 0.05‰.

The C_org_ concentrations were obtained by Rock–Eval pyrolysis following the procedure described by Behar et al.^100^, at the Institute of Earth Science of the University of Lausanne (ISTE-UNIL). The determined parameters are total organic carbon (TOC), the Hydrogen Index (HI as mg HC/g TOC) and the Oxygen Index (OI as mg CO_2_/g TOC), which permit an overall characterization of the sedimentary organic matter.

Mercury (Hg) content of the MC samples was measured with a Zeeman R-915F (Lumex, St-Petersburg, Russia) high-frequency atomic absorption spectrometer set at Mode 1 (700 °C) at the ISTE-UNIL. Analyses were performed on whole-rock powdered samples. The measurements were done in duplicates, and a certified external standard (GSD-11, Chinese alluvium, Hg: 72 ± 6 ppb) was used for calibration purposes (r = 0.99, for measured vs certified values) and to guarantee the analytical quality.

Micro-Raman analyses of iron oxides were conducted on six samples (MC1-33, MC2-31, MC3-10, MC3-33, MC3-37, MC3-60) at the LaSIE laboratory of La Rochelle University (France) using a Horiba High Resolution Raman spectrometer (LabRAM HR Evolution) equipped with a confocal microscope and a Peltier-based cooled charge coupled device (CCD) detector. Excitation was provided by a solid-state diode pumped green laser at 532 nm. The spot under the × 100 lens has a diameter of ~ 3 μm. Spectra were recorded with the acquisition LabSpec6 software at room temperature with a resolution of approximately 2 cm^−1^ and corresponded to an accumulation of 2 × 60 s acquisitions. The laser power was reduced to 0.03 mW in order to prevent the transformation of Fe-compounds into hematite (α-Fe_2_O_3_) due to an excessive heating.

Whole-rock mineralogy of MC samples was analyzed at the ISTE-UNIL by X-Ray Diffraction (XRD), using a Thermo Scientific ARL X-TRA diffractometer. The whole-rock mineralogy was determined by a semiquantitative method, using XRD peak intensities of the main minerals compared to external standards^101^.

For optical microscopy observations whole rock polished pellets of selected samples were prepared in accordance with standard procedures (ISO 7404-2, 2009). Samples were embedded in a mixture of resin and hardener and polished after drying using a Buehler grinder-polisher. Petrographic observations illustrated in Figure S6-D were carried out at Institute of Earth Sciences—Porto (Portugal) in a Leica 4000-M microscope equipped with a Discus-Fossil system under standard conditions, and a 50 × oil immersion objective.

## Supplementary Information

Supplementary Information.
