# Supplementary material for: Rapid light carbon releases and increased aridity linked to Karoo–Ferrar magmatism during the early Toarcian oceanic anoxic event
Source: Sci Rep. 2022 Mar 14;12:4342. doi: 10.1038/s41598-022-08269-y (PMC8921222; doi:10.1038/s41598-022-08269-y)
Supplement: Supplementary file 1 — Supplementary Information. [file 41598_2022_8269_MOESM1_ESM.pdf]

SUPPLEMENTARY MATERIALS

Rapid light carbon releases and increased aridity linked to Karoo-Ferrar magmatism during the early Toarcian oceanic anoxic event

Eric Font[1,2*], Luís Vítor Duarte[1,3], Mark J. Dekkers[4], Celine Remazeilles[5], Ramon Egli[6], Jorge E. Spangenberg[7], Alicia Fantasia[8], Joana Ribeiro[1,9], Elsa Gomes[1,10], José Mirão[11] and Thierry Adatte[12]

[1] University of Coimbra, Department of Earth Sciences, P-3030 790 Coimbra, Portugal,
[2] Instituto Dom Luís (IDL), Faculdade de Ciências, Universidade de Lisboa, 1749-026 Lisboa, Lisboa, Portugal
[3] University of Coimbra, MARE and Department of Earth Sciences, P-3030 790 Coimbra, Portugal,
[4] Paleomagnetic Laboratory 'Fort Hoofddijk', Department of Earth Sciences, Utrecht University, Princetonlaan 8a, 3584 CB Utrecht, The Netherlands
[5] Laboratoire des Sciences de l'Ingénieur pour l'Environnement, UMR CNRS 7356 La Rochelle Université, Pôle Sciences et Technologie, Avenue Michel Crépeau, 17042, La Rochelle Cedex 1, France. FR·
[6] Central Institute for Meteorology and Geodynamics (ZAMG), 1190 Wien, Austria AT [7] Institute of Earth Surface Dynamics, University of Lausanne, 1015 Lausanne, Switzerland
[8] Univ Lyon, UCBL, ENSL, UJM, CNRS, LGL-TPE, F-69622 Villeurbanne, France
[9] Institute of Earth Science - Porto Pol, Rua do campo alegre 687. 4169-007 Porto, Portugal
[10] CITEUC - Centre for Earth and Space Research of the University of Coimbra, 3040-004 Coimbra, Portugal
[11] HERCULES Laboratory, School of Sciences and Technology–Geosciences Department, University of Évora, 7000-671 Évora, Portugal
[12] ISTE, Geopolis, CH-1015 Lausanne, Switzerland

***Corresponding author:** Eric Font, e-mail: efont@uc.pt

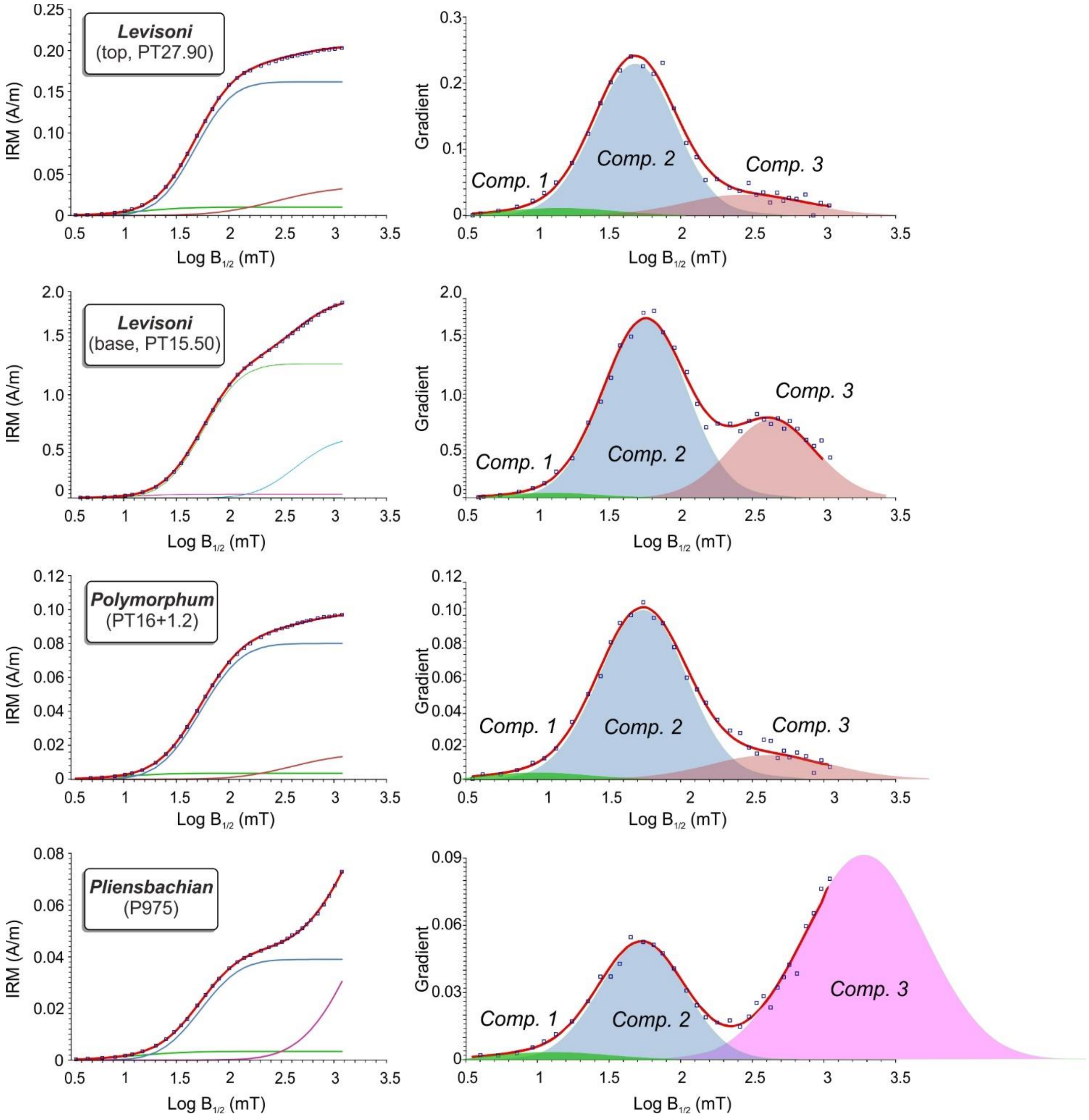

Figure S1. Isothermal remanent magnetization (IRM) acquisition curves of representative samples of the Peniche section (PT samples shown in Figure 2 in the main text) and corresponding output from the IRM coercivity analysis computed with the Kruiver, et al.[1] software (data in Table S2). Sample PT15.50 corresponds to the third brownish layer, the MC3 transect (see also main text Figures 1 and 3). Lower Toarcian sediments contain a mixture of magnetite (components 1 and 2) and hematite (component 3), whereas Pliensbachian sediments contain magnetite (components 1 and 2) and goethite (component 3).

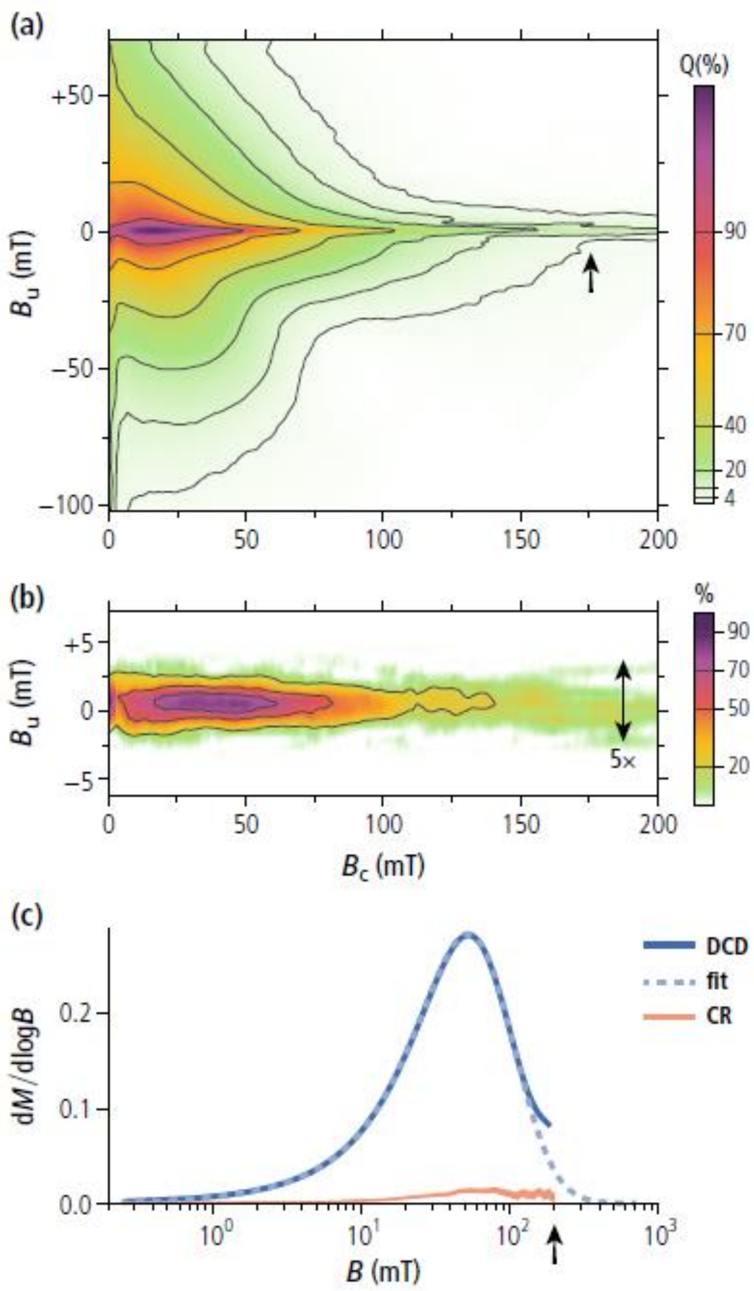

Figure S2. FORC characterization of sample 40 from MC3. (a) FORC diagram obtained with VARIFORC[2,3] using the following processing parameters: sc = su = 10, λ = 0.17, scr = 8, smoothing factor limit at coercive field: 20. Contour levels represent 4, 10, 20, 40, 70, and 90% quantiles of the total magnetization. The arrow at Bc ≈ 160 mT points to the termination of low-coercivity contributions associated with (titano-) magnetite. Beyond this limit, the contribution of hematite is visible as a continuation of the central ridge. (b) Central ridge[4] isolated from the FORC diagram in (a), which represents the contribution of non-interacting single-domain particles. Contour levels represent 20, 50, 70, and 90% of the maximum central ridge amplitude. Notice the 5× vertical exaggeration. (c) Coercivity distribution derived from a subset of FORC measurements corresponding to the Direct Current demagnetization (DCD) curve, and coercivity distribution of the central ridge. The dashed line represents a least-squares fit of the DCD curve with a skewed generalized Gaussian function[5,6]. The perfect overlap over the low coercivity range (left of the arrow) means that the DCD curve can be represented with a single coercivity component.

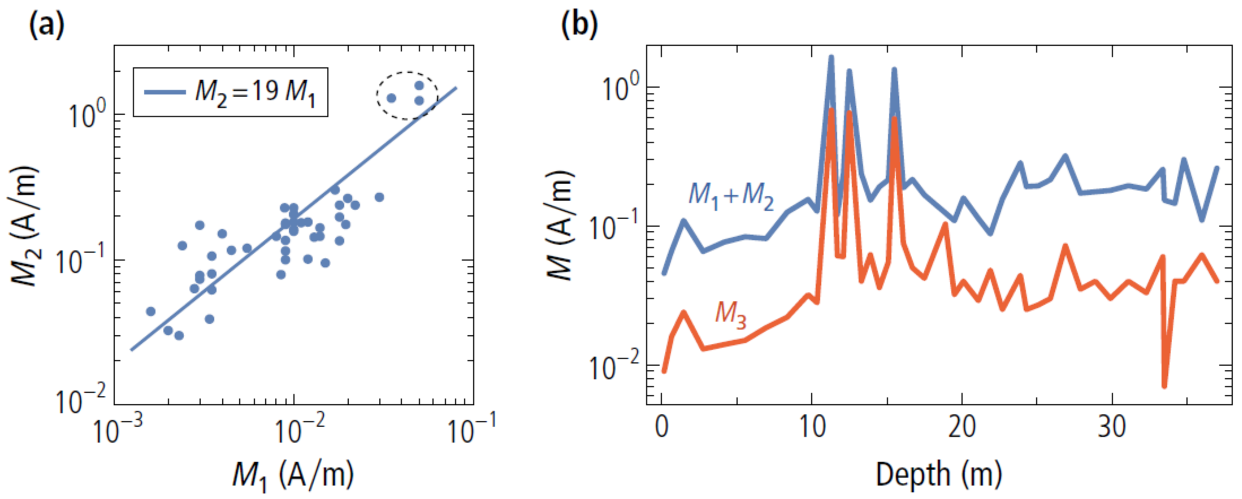

Figure S3. Scatter plot of the SIRM contributions M1 and M2 of components 1 and 2, respectively, obtained from Gaussian fits of IRM acquisition curves (Table S2). Circled points correspond to the three brown levels MC1, MC2, and MC3, respectively (note the bilogarithmic scale). The solid line indicates a proportionality relation between the two components. (b) Total SIRM contributions of magnetite (M1+M2) and hematite (M3) as a function of stratigraphic depth. Note the logarithmic scale.

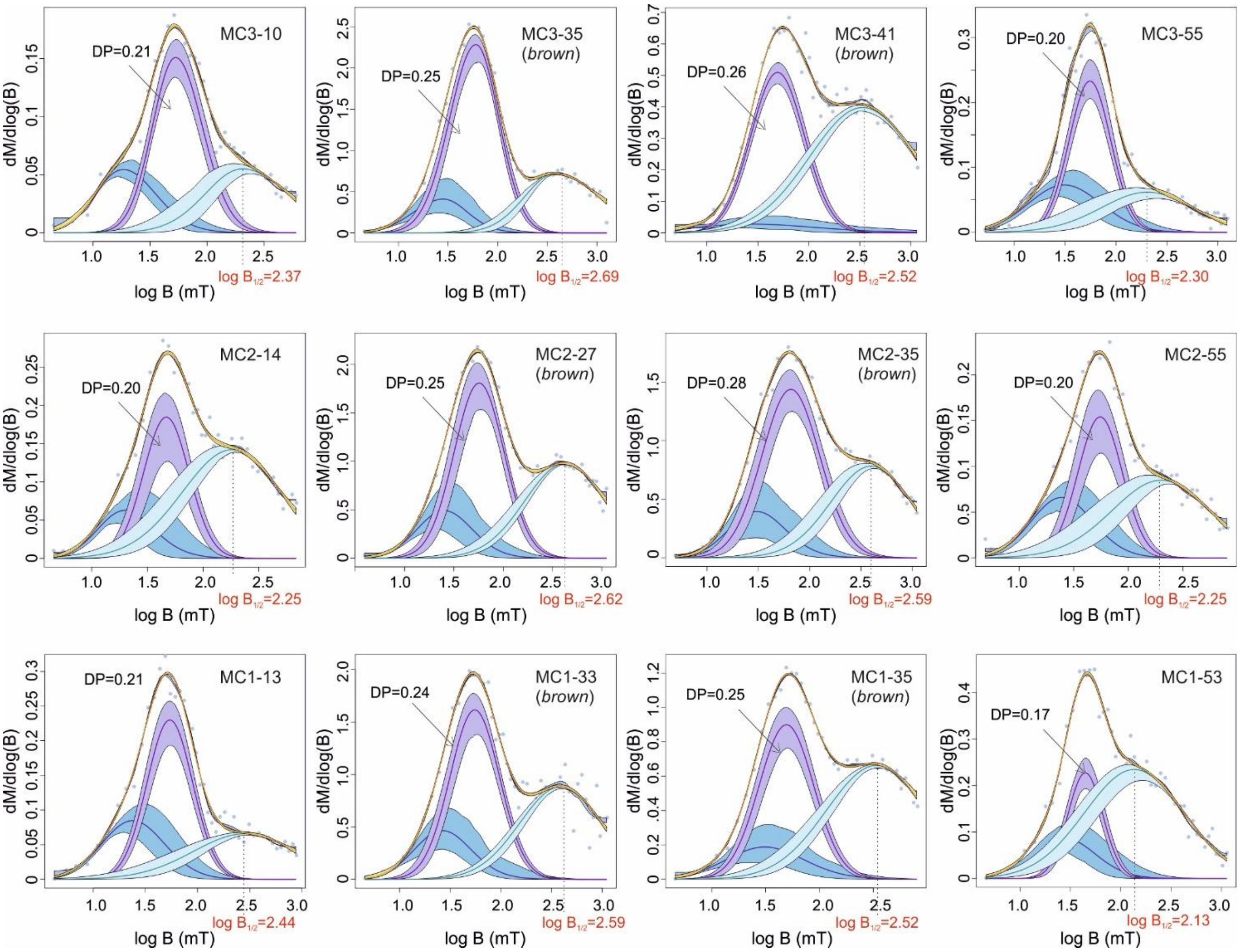

Figure S4. Coercivity distribution (data shown in grey circles, spline fit partially visible as black line) derived from IRM acquisition curve data of samples from MC1, MC2 and MC3, after unmixing with the Max Unmix software[7] (see also Figure 3). Shaded areas represent the 95% confidence interval uncertainty envelopes. Samples MC1-33, MC1-35, MC2-27, MC2-35, MC3-35 and MC3-41 correspond to the brownish intervals.

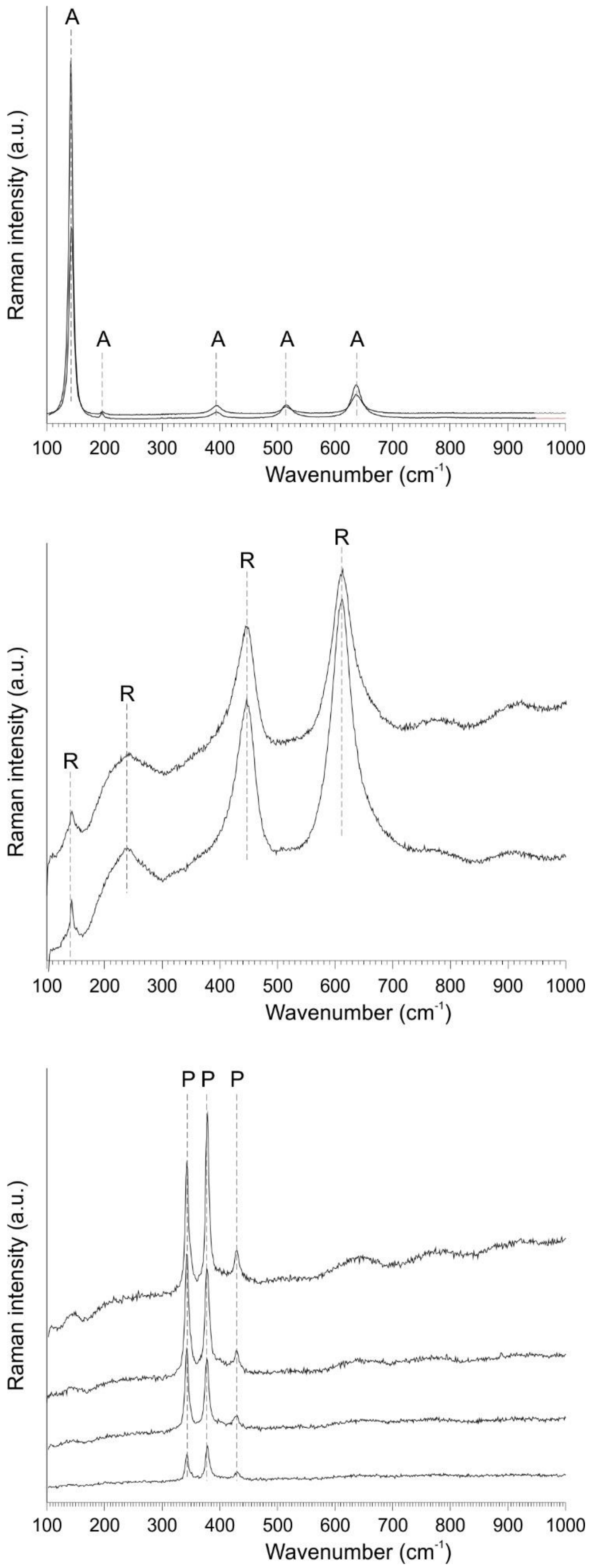

Figure S5. Representative micro-Raman spectra of MC samples (total of 6 samples). Anatase (A), rutile (R) and pyrite (P) are observed in all samples.

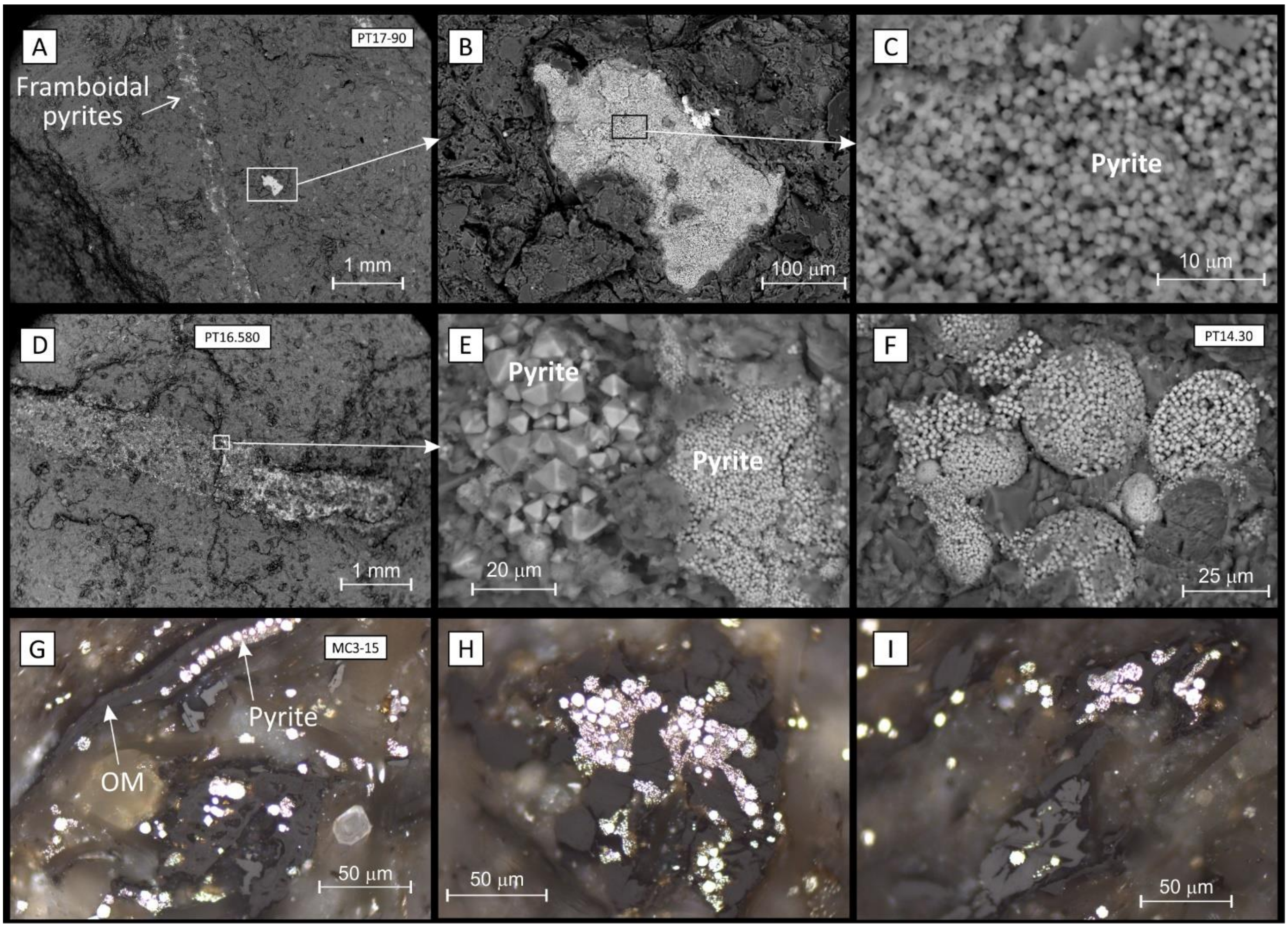

Figure S6. Illustrative (A-F) back-scattered Scanning Electron microscopic and (G-I) optical microscopic photographs under oil immersion of pyrite. Most of pyrite is represented by framboidal pyrite associated to organic matter.

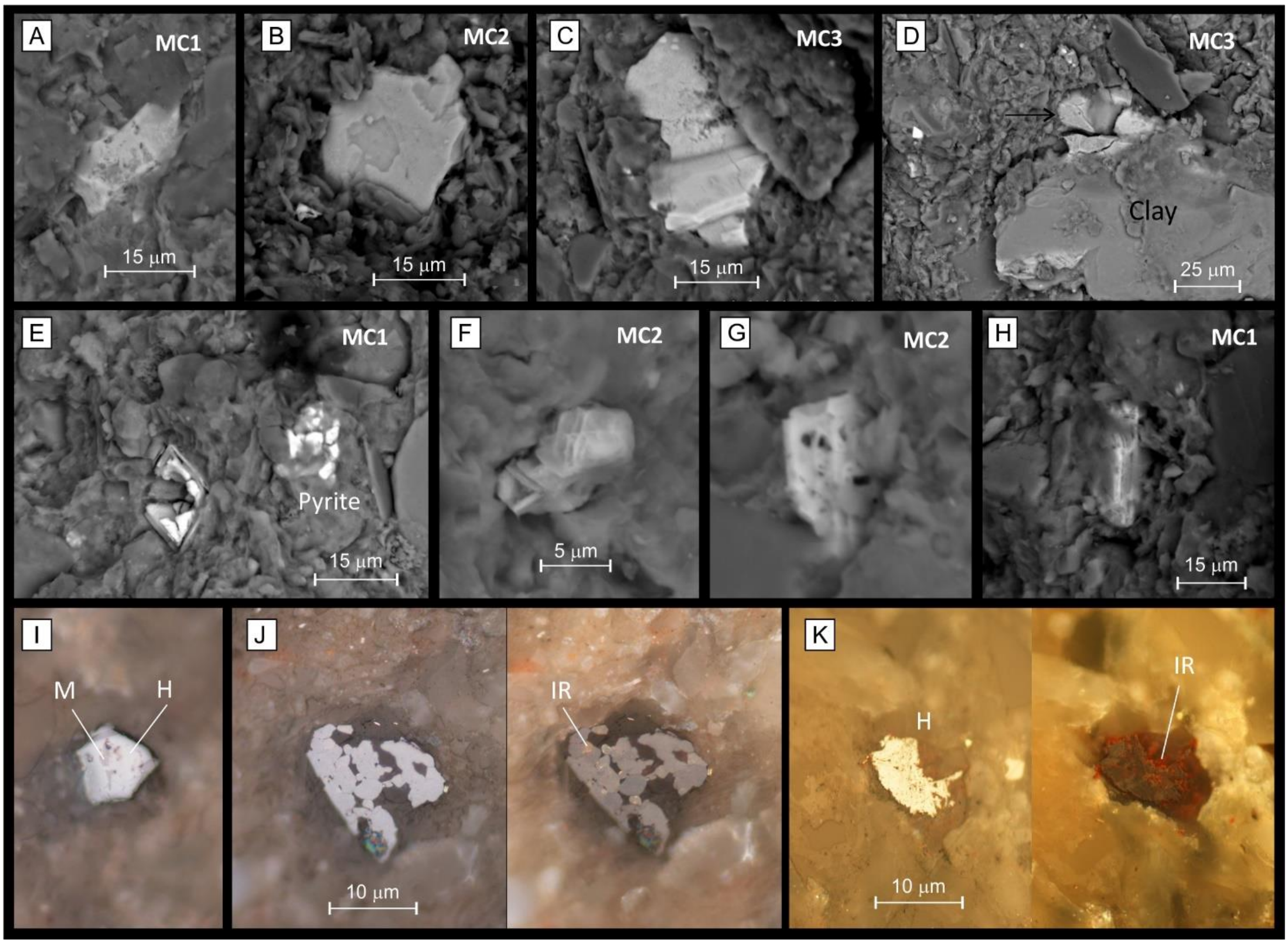

Figure S7. (A-H) Scanning Electron microscopic photographs of Ti-bearing iron oxides observed in the brown beds. Presence of Fe and Ti has been confirmed by Energy Dispersive Spectra (EDS) (not shown). Crystals exhibit different degree of alteration, from well preserved texture to dissolution and exsolution features. (I) Reflected light microphotograph of relics of magnetite in hematite (plane polarized light, sample MC3-33). (J) Reflected light microphotograph of martite, a pseudomorphic replacement of subhedral magnetite (M) to hematite (H), under plane polarized light (left) and crossed polarized light (right) (sample MC1-33). Red internal reflections (IR), typical of hematite, are observed and anisotropy can be distinguished by slight uncrossing the analyzer, in crossed polarized light. (K) Reflected light microphotograph of thin tabular hematite, under plane polarized light (left), and lamellar light and dark grey anisotropy and red internal reflections (IR), under crossed polarized light (right) (sample MC1-33).

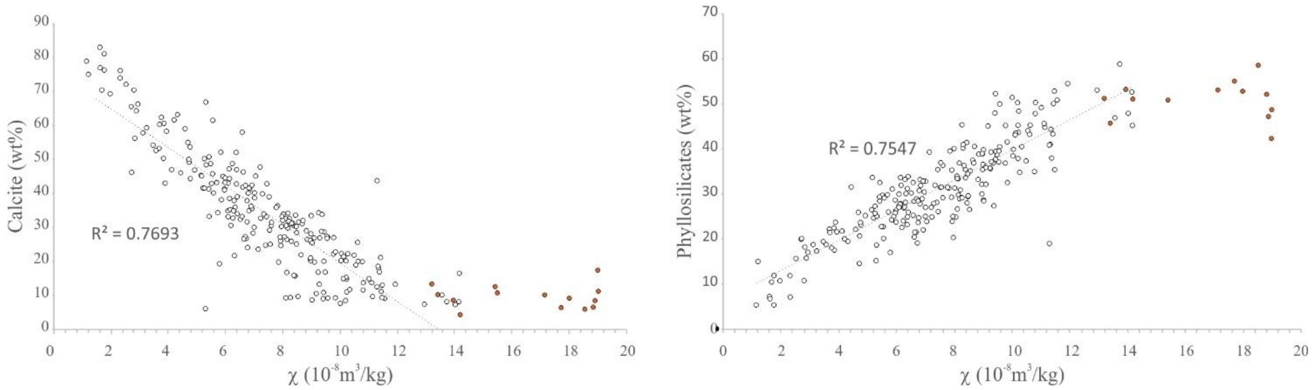

Figure S8. Correlation of magnetic susceptibility χ (this study) with the phyllosilicate and calcite content (data from Fantasia et al.[31] and this study) of the Peniche sediments (see data in Table S5). Brown circles correspond to the brownish layers. R^2 is the coefficient of determination and is calculated from the distribution of all samples excluding the brownish layers.

Sample	d (m)	MS (m3/kg)												
			PP16.140	7.78	5.42E-08	PT15,70	17.73	1.42E-07	PT25,50	27.53	6.57E-08	PPT35.50	37.53	7.44E-08
PPE962	0	2.31E-08	PP16.160	7.98	5.72E-08	PT15,90	17.93	1.14E-07	PT25,70	27.73	7.33E-08	PPT35.70	37.73	7.77E-08
PPE963	0.08	2.93E-08	PP16.180	8.18	5.96E-08	PT16,10	18.13	8.43E-08	PT25,90	27.93	5.98E-08	PPT35.90	37.93	8.21E-08
PPE964	0.14	2.80E-08	PP16.200	8.38	5.54E-08	PT16,30	18.33	1.14E-07	PT26,10	28.13	6.12E-08	PPT36.10	38.13	9.02E-08
PPE965	0.21	3.68E-08	PP16.220	8.58	5.42E-08	PT16,50	18.53	1.05E-07	PT26,30	28.33	9.84E-08	PPT36.30	38.33	8.11E-08
PPE966	0.27	1.60E-08	PP16.240	8.78	4.58E-08	PT16,70	18.73	1.00E-07	PT26,50	28.53	8.48E-08	PPT36.50	38.53	5.48E-08
PPE967	0.33	2.70E-08	PP16.260	8.98	4.20E-08	PT16,90	18.93	1.13E-07	PT26,70	28.73	7.29E-08	PPT36.70	38.73	6.18E-08
PPE968	0.38	1.20E-08	PP16.280	9.18	4.92E-08	PT17,10	19.13	1.00E-07	PT26,90	28.93	7.97E-08	PPT36.90	38.93	6.96E-08
PPE969	0.48	3.24E-08	PP16.300	9.38	4.68E-08	PT17,30	19.33	9.23E-08	PT27,10	29.13	1.02E-07	PPT37.10	39.13	6.62E-08
PPE970	0.53	1.62E-08	PP16.320	9.58	6.40E-08	PT17,50	19.53	6.58E-08	PT27,30	29.33	8.47E-08			
PPE971	0.6	2.88E-08	PP16.340	9.78	5.32E-08	PT17,70	19.73	1.06E-07	PT27,50	29.53	5.55E-08		D1 (see Fig. 2)	
PPE972	0.64	1.14E-08	PP16.360	9.98	5.98E-08	PT17,90	19.93	1.06E-07	PT27,70	29.73	7.21E-08		D3	
PPE973	0.72	2.83E-08	PP16.380	10.18	6.38E-08	PT18,10	20.13	1.06E-07	PT27,90	29.93	6.70E-08		Brownish levels	
PPE974	0.82	1.68E-08	PP16.400	10.38	6.79E-08	PT18,30	20.33	1.01E-07	PT28,10	30.13	6.82E-08			
PPE975	0.92	3.69E-08	PP16.420	10.58	6.33E-08	PT18,50	20.53	8.99E-08	PT28,30	30.33	8.24E-08			
PPE976	1.02	2.32E-08	PP16.440	10.78	5.69E-08	PT18,70	20.73	9.35E-08	PT28,50	30.53	8.05E-08			
PPE977	1.12	2.71E-08	PP16.460	10.98	5.26E-08	PT18,90	20.93	8.47E-08	PT28,70	30.73	7.00E-08			
PPE978	1.28	2.52E-08	PP16.480	11.18	6.25E-08	PT19,10	21.13	8.39E-08	PT28,90	30.93	7.61E-08			
PPE979	1.49	3.57E-08	PP16.500	11.38	6.28E-08	PT19,30	21.33	1.13E-07	PT29,10	31.13	4.71E-08			
PPE980	1.6	1.96E-08	PP16.520	11.58	8.30E-08	PT19,50	21.53	1.02E-07	PPT29.3	31.33	1.08E-07			
PPE981	1.67	3.83E-08	PP16.540	11.78	7.43E-08	PT19,75	21.73	8.96E-08	PPT29.50	31.53	8.82E-08			
PPE982	1.77	1.76E-08	PP16.560	11.98	6.51E-08	PT19,90	21.93	9.10E-08	PPT29.70	31.73	5.78E-08			
PPE983	1.83	3.46E-08	PP16.580	12.18	5.90E-08	PT20,10	22.13	8.64E-08	PPT29.90	31.93	7.56E-08			
PPE984	2.08	1.76E-08	PP16.600	12.38	6.24E-08	PT20,30	22.33	7.85E-08	PPT30.10	32.13	8.44E-08			
PP1	2.18	5.31E-08	PP16.620	12.58	7.01E-08	PT20,50	22.53	7.52E-08	PPT30.30	32.33	9.54E-08			
PP2	2.28	4.72E-08	PP16.640	12.78	1.20E-07	PT20,70	22.73	8.98E-08	PPT30.50	32.53	8.96E-08			
PP3	2.43	7.60E-08	PP16.660	12.98	1.41E-07	PT20,90	22.93	9.01E-08	PPT30.70	32.73	7.90E-08			
PP4	2.58	6.08E-08	PT11.30	13.33	1.67E-07	PT21,10	23.13	8.14E-08	PPT30.90	32.93	6.72E-08			
PP5	2.68	5.51E-08	PT11,50	13.53	1.02E-07	PT21,30	23.33	8.39E-08	PPT31.10	33.13	6.69E-08			
PP6	2.78	3.89E-08	PT11,70	13.73	9.48E-08	PT21,55	23.53	7.14E-08	PPT31.30	33.33	8.18E-08			
PP7	2.88	4.12E-08	PT11,90	13.93	9.77E-08	PT21,60	23.63	5.83E-08	PPT31.50	33.53	7.05E-08			
PP8	3.18	4.32E-08	PT12,10	14.13	9.78E-08	PT21,70	23.73	7.82E-08	PPT31.70	33.73	7.93E-08			
PP9	3.33	5.14E-08	PT12,30	14.33	9.25E-08	PT21,90	23.93	7.92E-08	PPT31.90	33.93	9.43E-08			
PP10	3.48	4.77E-08	PT12,50	14.53	1.88E-07	PT22,10	24.13	7.39E-08	PPT32.10	34.13	8.97E-08			
PP11	3.58	5.41E-08	PT12,70	14.73	1.16E-07	PT22,30	24.33	8.98E-08	PPT32.30	34.33	1.03E-07			
PP12	3.98	3.85E-08	PT12,90	14.93	9.57E-08	PT22,50	24.53	9.10E-08	PPT32.50	34.53	8.27E-08			
PP13INF	4.23	6.81E-08	PT13,10	15.13	1.10E-07	PT22,70	24.73	5.26E-08	PPT32.70	34.73	7.00E-08			
PP13MID	4.43	6.13E-08	PT13,30	15.33	1.08E-07	PT22,90	24.93	5.85E-08	PPT32.90	34.93	6.41E-08			
PP13SUP	4.63	6.16E-08	PPT13.45	15.48	1.11E-07	PT23,10	25.13	8.09E-08	PPT33.10	35.13	6.89E-08			
PP14	4.98	3.11E-08	PT13,50	15.53	5.29E-08	PT23,30	25.33	8.17E-08	PPT33.30	35.33	6.92E-08			
PP15INF	5.33	3.93E-08	PT13,70	15.73	8.53E-08	PT23,50	25.53	6.38E-08	PPT33.50	35.53	6.09E-08			
PP15MID	5.63	4.43E-08	PT13,90	15.93	9.02E-08	PT23,70	25.73	9.57E-08	PPT33.70	35.73	6.75E-08			
PP15SUP	5.93	6.09E-08	PT14,10	16.13	9.44E-08	PT23,90	25.93	8.85E-08	PPT33.80	35.83	6.31E-08			
PP16	6.38	3.76E-08	PT14,30	16.33	8.56E-08	PT24,10	26.13	7.99E-08	PPT34.10	36.13	7.53E-08			
PP16.20	6.58	5.17E-08	PT14,50	16.53	9.37E-08	PT24,30	26.33	6.84E-08	PPT34.30	36.33	6.75E-08			
PP16.40	6.78	5.21E-08	PT14,70	16.73	1.14E-07	PT24,50	26.53	8.67E-08	PPT34.50	36.53	7.95E-08			
PP16.60	6.98	4.76E-08	PT14,90	16.93	1.15E-07	PT24,70	26.73	8.02E-08	PPT34.70	36.73	6.77E-08			
PP16.80	7.18	6.29E-08	PT15,10	17.13	1.13E-07	PT24,90	26.93	6.37E-08	PPT34.90	36.93	5.96E-08			
PP16.100	7.38	5.58E-08	PT15,30	17.33	1.13E-07	PT25,10	27.13	6.92E-08	PPT35.10	37.13	7.23E-08			
PP16.120	7.58	6.14E-08	PT15,50	17.53	1.90E-07	PT25,30	27.33	6.60E-08	PPT35.30	37.33	9.29E-08			

Table S1. Mass specific magnetic susceptibility of the Peniche samples collected by Fantasia et al.[8]. Brown beds are highlighted in grey.

		Comp. 1 (<20 mT)				Comp. 2 (30-60 mT)				Comp. 3 (150-450 mT)				S-ratio
Samples	d (cm)	%	SIRM (A/m)	$B_{1/2}$	DP	%	SIRM (A/m)	$B_{1/2}$	DP	%	SIRM (A/m)	$B_{1/2}$	DP	(calculado)
P960	-2.30	3	0.0023	10.0	0.40	40	0.0300	53.7	0.31	57	0.0430	1778.3	0.50	0.52
P968	-1.65	2	0.0020	10.0	0.40	32	0.0325	53.7	0.30	66	0.0670	1778.3	0.50	0.39
P975	-1.11	2	0.0034	12.6	0.40	27	0.0390	53.7	0.30	70	0.1000	1995.3	0.44	0.35
P982	0.15	3	0.0016	10.0	0.35	81	0.0440	55.0	0.32	16	0.0090	398.1	0.40	0.83
P5	0.65	3	0.0028	10.0	0.35	77	0.0632	55.0	0.33	20	0.0160	331.1	0.40	0.81
P10	1.45	3	0.0035	10.0	0.35	79	0.1060	58.9	0.34	18	0.0240	316.2	0.38	0.81
P13T	2.75	4	0.0035	10.0	0.32	79	0.0620	56.2	0.31	17	0.0130	354.8	0.39	0.83
P15T	4.15	3	0.0030	10.0	0.35	81	0.0734	53.7	0.31	15	0.0140	354.8	0.37	0.84
P16+1,2	5.55	4	0.0035	10.0	0.35	81	0.0800	53.7	0.31	15	0.0150	398.1	0.40	0.84
P16+2,6	6.95	3	0.0030	10.0	0.35	78	0.0780	53.7	0.30	19	0.0185	354.8	0.45	0.83
P16+4,0	8.35	4	0.0055	10.0	0.35	81	0.1200	52.5	0.31	15	0.0220	398.1	0.45	0.86
P16+5,4	9.75	2	0.0040	10.0	0.30	81	0.1510	57.5	0.33	17	0.0320	407.4	0.45	0.83
P16+6,8	10.35	2	0.0024	10.0	0.30	80	0.1250	53.7	0.33	18	0.0280	316.2	0.40	0.83
PT11.30	11.3	2	0.0500	12.6	0.33	69	1.5900	56.2	0.29	29	0.6800	416.9	0.30	0.64
PT11.70	11.7	2	0.0045	12.6	0.33	64	0.1162	56.2	0.33	34	0.0610	281.8	0.37	0.71
PT12.10	12.1	3	0.0100	12.6	0.35	77	0.2280	50.1	0.30	20	0.0600	354.8	0.30	0.78
PT12.50	12.5	3	0.0500	12.6	0.30	64	1.2500	56.2	0.29	33	0.6500	416.9	0.35	0.63
PT13.30	13.3	3	0.0089	12.6	0.30	82	0.2280	47.9	0.27	14	0.0400	354.8	0.38	0.86
PT13.90	13.9	4	0.0080	11.2	0.30	67	0.1450	53.7	0.31	29	0.0620	302.0	0.40	0.75
PT14.50	14.5	5	0.0110	12.6	0.35	79	0.1800	50.1	0.30	16	0.0360	354.8	0.40	0.85
PT15.10	15.1	4	0.0100	12.6	0.30	76	0.2050	47.9	0.28	20	0.0550	316.2	0.35	0.81
PT15.50	15.5	2	0.0350	12.6	0.30	68	1.3000	56.2	0.29	31	0.5900	426.6	0.30	0.62
PT16.10	16.1	3	0.0090	12.6	0.35	68	0.1790	47.9	0.31	29	0.0750	316.2	0.38	0.74
PT16.70	16.7	4	0.0100	12.6	0.33	77	0.2060	47.9	0.28	19	0.0500	316.2	0.40	0.84
PT17.50	17.5	5	0.0100	12.6	0.35	75	0.1570	50.1	0.29	20	0.0420	316.2	0.33	0.81
PT18.90	18.90	4	0.0090	14.1	0.35	51	0.1150	46.8	0.28	45	0.1030	245.5	0.40	0.67
PT19.50	19.50	6	0.0090	12.6	0.30	71	0.1000	47.9	0.28	23	0.0320	316.2	0.45	0.82
PT20.10	20.10	7	0.0140	14.1	0.35	73	0.1450	47.9	0.27	20	0.0400	316.2	0.45	0.84
PT21.10	21.10	8	0.0120	14.8	0.30	71	0.1010	47.9	0.26	20	0.0290	309.0	0.43	0.83
PT21.90	21.90	6	0.0085	12.6	0.30	58	0.0790	49.0	0.27	35	0.0480	208.9	0.38	0.78
PT22.70	22.70	7	0.0130	14.1	0.30	79	0.1430	47.9	0.27	14	0.0250	316.2	0.40	0.88
PT23.90	23.90	6	0.0200	14.1	0.30	81	0.2650	44.7	0.24	13	0.0440	234.4	0.40	0.91
PT24.30	24.30	5	0.0100	14.1	0.33	84	0.1820	46.8	0.26	12	0.0250	380.2	0.45	0.90
PT25.10	25.10	5	0.0120	14.1	0.33	82	0.1820	46.8	0.27	12	0.0270	380.2	0.45	0.89
PT25.90	25.90	7	0.0180	14.1	0.33	80	0.1970	49.0	0.27	12	0.0300	398.1	0.44	0.89
PT26.90	26.90	4	0.0170	12.6	0.28	77	0.3030	42.7	0.22	18	0.0720	182.0	0.45	0.90
PT27.90	27.90	5	0.0100	14.1	0.35	78	0.1620	47.9	0.28	17	0.0350	281.8	0.45	0.87
PT28.90	28.90	1	0.0030	12.6	0.30	79	0.1730	45.7	0.27	18	0.0400	251.2	0.50	0.88
PT29.90	29.90	7	0.0140	12.6	0.30	79	0.1660	47.9	0.27	14	0.0300	251.2	0.50	0.90
PT31.10	31.10	8	0.0195	15.8	0.28	75	0.1750	47.9	0.26	17	0.0400	316.2	0.40	0.85
PT32.30	32.30	4	0.0090	12.6	0.28	81	0.1750	47.9	0.28	15	0.0330	316.2	0.45	0.88
PT33.40	33.40	6	0.0180	14.1	0.28	75	0.2380	46.8	0.26	19	0.0600	251.2	0.40	0.86
PT33.50	33.50	11	0.0180	14.1	0.28	84	0.1350	50.1	0.26	4	0.0070	446.7	0.30	0.95
PT34.20	34.20	5	0.0090	12.6	0.30	74	0.1360	52.5	0.28	22	0.0400	281.8	0.28	0.81
PT34.80	34.80	9	0.0300	12.6	0.25	79	0.2700	50.1	0.28	12	0.0400	281.8	0.45	0.91
PT36.00	36.00	9	0.0150	15.8	0.30	55	0.0950	50.1	0.26	36	0.0620	199.5	0.35	0.79
PT37.00	37.00	7	0.0220	12.6	0.24	79	0.2380	51.3	0.27	13	0.0400	281.8	0.40	0.89
	Brownish levels													

Table S2. IRM component fitting parameters of the Peniche sample calculated using the Kruiver et al.[1] software. SIRM is the saturation isothermal remanent magnetization; B1/2 (mT) is the field at which half of the SIRM is reached; DP is the dispersion parameter (i.e., width of the distribution) in log field values. The forward S-ratio is calculated based on the formula -IRM-0.3T/IRM1T. Brownish layers are highlighted in grey. The fits are illustrated in Figure S1 and relevant parameters plotted in Figure 2 of the main text.

			Comp. 1				Comp. 2				Comp. 3				
Samples	d(cm)	χ (10^{-8} m^3/kg)	%	SIRM (A/m)	$B_{1/2}$	DP	%	SIRM (A/m)	$B_{1/2}$	DP	%	SIRM (A/m)	$B_{1/2}$	DP	S-ratio
MC1-	0	8.14													
MC1-	3	7.10													
MC1-	5	6.63													
MC1-	8	8.69													
MC1-	11	9.27													
MC1-	13	10.01	23	0.06	21	0.26	53	0.14	56	0.21	24	0.06	282	0.35	0.80
MC1-	16	10.34													
MC1-	19	9.83	13	0.05	20	0.30	56	0.19	52	0.24	31	0.11	263	0.29	0.75
MC1-	20	10.26													
MC1-	23	11.13													
MC1-	25	10.43	25	0.05	20	0.28	57	0.38	52	0.26	36	0.24	331	0.34	0.65
MC1-	27	13.20													
MC1-	29	13.40	7	0.12	20	0.30	57	1.00	54	0.26	36	0.62	372	0.36	0.64
MC1-	33	15.48	6	0.14	20	0.30	58	1.25	52	0.26	36	0.78	398	0.35	0.61
MC1-	35	14.20	10	0.14	21	0.26	45	0.65	52	0.24	46	0.67	339	0.38	0.57
MC1-	38	10.93	9	0.08	22	0.30	55	0.50	56	0.26	37	0.34	363	0.33	0.61
MC1-	42	9.17	9	0.03	20	0.28	30	0.10	54	0.23	61	0.21	234	0.34	0.57
MC1-	44	9.42													
MC1-	48	9.91	51	0.05	20	0.28	51	0.23	49	0.22	38	0.18	234	0.32	0.73
MC1-	53	8.27	12	0.06	21	0.28	49	0.22	50	0.22	39	0.17	224	0.33	0.74
MC2-	0	10.01													
MC2-	2	8.89													
MC2-	5	8.10	15	0.03	21	0.26	40	0.07	55	0.25	45	0.07	224	0.36	0.70
MC2-	7	9.19													
MC2-	9	9.35													
MC2-	10	9.41													
MC2-	12	9.67													
MC2-	14	10.19	10	0.03	20	0.30	48	0.14	47	0.24	41	0.12	224	0.35	0.73
MC2-	16	10.67													
MC2-	18	11.47	25	0.05	19	0.30	49	0.27	49	0.26	43	0.24	316	0.35	0.60
MC2-	20	12.94													
MC2-	21	13.56													
MC2-	23	14.02	8	0.10	20	0.35	54	0.70	55	0.26	38	0.50	398	0.36	0.59
MC2-	25	17.13													
MC2-	27	17.71	8	0.20	20	0.30	56	1.35	55	0.27	35	0.85	398	0.35	0.62
MC2-	29	18.46													
MC2-	31	18.53	9	0.23	20	0.35	52	1.27	50	0.26	39	0.95	398	0.36	0.59
MC2-	33	16.62													
MC2-	35	14.19	6	0.12	22	0.35	57	1.17	60	0.28	37	0.75	380	0.36	0.61
MC2-	37	14.27													
MC2-	39	13.73													
MC2-	41	11.93	10	0.07	20	0.30	46	0.32	52	0.24	43	0.30	331	0.37	0.59
MC2-	43	9.65	52	0.04	20	0.30	52	0.13	55	0.27	35	0.09	302	0.33	0.68
MC2-	45	8.41													
MC2-	46	8.34													
MC2-	48	8.88													
MC2-	50	8.70													
MC2-	52	8.99													
MC2-	55	8.31	18	0.04	21	0.30	49	0.11	56	0.23	33	0.07	251	0.35	0.75
MC2-	58	9.55													
MC2-	62	9.40													
MC2-	64	9.92													
MC2-	67	8.17													
MC2-	70	7.08	23	0.06	21	0.26	53	0.14	56	0.21	24	0.06	282	0.35	0.80
MC3-	0	10.23	24	0.045	22	0.26	47	0.090	54	0.19	29	0.055	224	0.30	0.81
MC3-	5	10.99													
MC3-	10	10.97	26	0.045	21	0.27	40	0.069	58	0.20	34	0.058	200	0.40	0.80
MC3-	15	11.11													
MC3-	20	11.40	18	0.060	23	0.25	43	0.142	56	0.21	38	0.125	295	0.35	0.67
MC3-	25	15.41	8	0.060	20	0.30	64	0.500	51	0.26	29	0.226	398	0.37	0.70
MC3-	27	16.78													
MC3-	28	17.39	10	0.200	21	0.26	64	1.270	56	0.24	27	0.530	398	0.33	0.71
MC3-	29	17.63													
MC3-	30	17.99													
MC3-	31	18.96	12	0.260	23	0.23	58	1.270	60	0.21	30	0.650	398	0.36	0.69
MC3-	33	18.70													
MC3-	35	19.00	21	0.500	28	0.25	49	1.160	60	0.21	29	0.690	389	0.37	0.70
MC3-	37	18.23													
MC3-	38	19.71	10	0.280	22	0.24	59	1.600	60	0.22	30	0.820	398	0.37	0.69
MC3-	40	18.89													
MC3-	41	13.95	4	0.030	22	0.22	54	0.450	56	0.28	42	0.350	380	0.34	0.54
MC3-	42	12.96													
MC3-	43	11.93													
MC3-	44	11.97													
MC3-	45	11.62													
MC3-	47	11.81	15	0.045	20	0.26	35	0.108	50	0.20	51	0.157	209	0.40	0.69
MC3-	50	10.81													
MC3-	55	10.49	18	0.045	21	0.26	56	0.140	55	0.19	26	0.066	200	0.35	0.85
MC3-	60	10.53													
MC3-	62	10.50													
MC3-	65	10.17	16	0.033	21	0.26	43	0.090	50	0.21	41	0.085	178	0.45	0.78
MC3-	70	10.11													

Table S3. Mass specific magnetic susceptibility (χ) and IRM component parameters after unmixing using the Kruiver et al.[1] software of the brown beds (MC samples). SIRM is the saturation isothermal remanent magnetization; % is the contribution of the magnetic component in percentage; $B_{1/2}$ (mT) is the field at which half of the SIRM is reached; DP is the dispersion parameter (i.e., width of the distribution) in log field values. The forward S-ratio is calculated based on the formula -IRM-0.3T/IRM1T. Parameters are plotted in Figure 3. Fits using the Maxunmix[7] software are shown in Figure S3. Both software packages provide similar fits.

Sample	$\delta^{13}C_{org}$ (VPDB)	TOC (%)	HI	OI	Hg (ppb)	Hg(ppb)/TOC(%)
MC1-0	-26.77	0.34	61.09	116.24	0.337	90.034
MC1-5	-26.50	0.26	40.15	82.29	0.256	133.006
MC1-11	-26.58	0.27	57.91	71.62	0.267	85.031
MC1-13		0.26	53.10	140.22	0.260	95.756
MC1-16	-26.53	0.24	50.59	181.21	0.245	124.620
MC1-19	-26.56	0.22	41.93	81.95	0.222	138.445
MC1-23	-26.74	0.25	40.94	592.30	0.253	94.194
MC1-25	-26.28	0.24	50.84	678.33	0.243	90.975
MC1-27	-26.29	0.24	51.91	584.16	0.238	122.699
MC1-29	-26.35	0.21	55.13	426.81	0.205	144.575
MC1-33	-26.41	0.20	36.69	175.38	0.172	220.909
MC1-35	-25.51	0.16	0.00	24.60	0.160	268.497
MC1-38	-25.19	0.17	35.89	45.65	0.196	240.262
MC1-42	-25.49	0.29	42.76	30.56	0.289	107.537
MC1-44	-25.79	0.27	49.12	161.89	0.273	94.587
MC1-48		0.29	33.44	70.08	0.295	91.076
MC1-53	-25.13	0.25	32.42	34.45	0.255	141.337
MC2-0	-27.98	0.35	49.73	38.92	41.30	117.18
MC2-5	-28.33	0.31	56.73	57.02	30.80	99.66
MC2-10	-28.14	0.27	49.24	125.72	59.60	218.86
MC2-14	-28.61	0.33	43.61	47.22	34.50	104.18
MC2-18	-28.73	0.28	47.91	31.39	57.80	206.42
MC2-20	-28.58	0.26	56.57	30.56	43.50	170.31
MC2-21	-28.46	0.28	40.23	38.00	39.00	138.16
MC2-23	-28.91	0.21	39.17	64.62	59.10	280.19
MC2-25	-28.12	0.20	47.43	49.64	51.10	260.75
MC2-27	-28.48	0.15	46.71	31.39	50.60	326.81
MC2-31	-27.67	0.16	50.46	59.28	82.60	504.18
MC2-35	-27.55	0.25	37.94	39.59	57.10	232.93
MC2-39	-26.54	0.39	34.47	34.82	85.70	219.81
MC2-41	-26.11	0.35	38.42	44.31	66.90	191.22
MC2-43	-26.04	0.29	49.48	281.26	14.70	50.24
MC2-46	-25.98	0.28	47.40	92.06	17.80	62.74
MC2-50	-26.53	0.23	58.63	88.49	17.30	75.42
MC2-55	-26.23	0.27	49.63	110.46	16.80	61.98
MC2-64		0.23	40.70	75.91	18.00	78.66
MC2-70		0.16	50.46	99.87	17.80	108.45
MC3-0		0.55	89.00	153.00	0.55	58.55
MC3-5		0.47	56.85	160.33	0.47	55.11
MC3-10	-29.92	0.49	50.17	147.91	0.49	67.14
MC3-15		0.39	111.00	187.00	0.39	65.90
MC3-20	-29.54	0.34	37.73	167.58	0.34	61.61
MC3-25	-29.47	0.32	123.00	100.00	0.32	130.00
MC3-27		0.32	36.80	218.57	0.32	135.03
MC3-28	-29.65	0.29	31.00	152.44	0.29	158.53
MC3-29		0.28	39.07	139.75	0.28	203.37
MC3-30	-29.81	0.28	140.00	149.00	0.28	153.93
MC3-31		0.25	35.91	210.31	0.25	129.58
MC3-33	-29.80	0.27	36.71	238.57	0.27	143.89
MC3-35	-29.85	0.31	127.00	211.00	0.31	118.39
MC3-37		0.25	43.44	373.03	0.25	181.76
MC3-38	-30.00	0.27	36.58	350.13	0.27	163.06
MC3-40		0.26	143.00	243.00	0.26	231.92
MC3-41		0.28	130.00	302.00	0.28	172.86
MC3-42	-29.62	0.26	34.80	213.62	0.26	250.95
MC3-43		0.28	31.47	197.61	0.28	246.18
MC3-44		0.21	33.13	331.57	0.21	388.64
MC3-45	-29.05	0.31	35.17	243.75	0.31	230.15
MC3-47		0.59	31.74	141.79	0.59	110.57
MC3-50	-28.86	0.53	97.00	138.00	0.53	98.87
MC3-55		0.50	39.78	63.64	0.50	57.49
MC3-60	-28.77	0.44	42.76	38.79	0.44	50.16
MC3-62		0.43	38.86	39.42	0.43	55.02
MC3-70		0.54	85.00	19.00	0.54	55.00

Table S4. Carbon isotope composition ($\delta^{13}C_{org}$), total organic carbon (TOC), OI (oxygen index) and HI (hydrogen index), mercury (Hg) and Hg/TOC ratio of the brown beds (MC samples). Data are shown in Figure 3.

Sample	d (m)	Phyllosilicates (%)	Quartz (%)	Feldspath-K (%)	Plagioclase-Na (%)	Calcite (%)	Dolomite (%)	Pyrite (%)	Goethite (%)	Gypse (%)	Ankérite (%)	Indosés (%)	χ (m^3/kg)
Data from Fantasia et al. (2019)													
PPE962	-2.03	11.84	8.84	0.38	3.17	74.00	0.00	0.00	0.00	0.00	0.00	1.77	2.31E-08
PPE963	-1.95	17.05	10.27	0.54	3.32	66.34	0.00	0.00	0.00	0.00	0.00	2.48	2.93E-08
PPE964	-1.89	10.78	11.45	0.74	3.38	70.45	0.00	0.00	0.00	0.00	0.00	3.20	2.80E-08
PPE965	-1.82	22.19	15.78	0.87	5.05	53.29	0.00	0.00	0.00	0.00	0.00	2.82	3.68E-08
PPE966	-1.76	7.24	5.49	0.00	3.37	83.05	0.00	0.00	0.00	0.00	0.00	0.84	1.60E-08
PPE967	-1.7	19.93	8.65	1.73	2.21	65.60	0.00	0.00	0.00	0.00	0.00	1.89	2.70E-08
PPE968	-1.65	15.00	5.45	0.00	3.42	75.07	0.00	0.00	0.00	0.00	0.00	1.06	1.20E-08
PPE969	-1.55	17.31	16.34	0.00	4.67	59.34	0.00	0.00	0.00	0.00	0.00	2.34	3.24E-08
PPE970	-1.5	6.72	6.24	0.00	7.47	77.03	0.29	0.00	0.00	0.00	0.00	2.24	1.62E-08
PPE971	-1.43	15.78	13.67	0.00	2.94	64.34	0.00	0.00	0.00	0.00	0.00	3.27	2.88E-08
PPE972	-1.39	5.34	7.34	0.00	5.53	78.98	0.00	0.00	0.00	0.00	0.00	2.81	1.14E-08
PPE973	-1.31	18.20	15.25	2.18	5.37	56.23	0.00	0.00	0.00	0.00	0.00	2.76	2.83E-08
PPE974	-1.21	10.45	13.67	0.00	3.29	70.45	0.00	0.00	0.00	0.00	0.00	2.14	1.68E-08
PPE975	-1.11	21.45	11.65	1.69	3.42	60.34	0.00	0.00	0.00	0.00	0.00	1.45	3.69E-08
PPE976	-1.01	7.11	11.10	0.38	4.21	76.18	0.00	0.00	0.00	0.00	0.00	1.02	2.32E-08
PPE977	-0.91	20.12	28.45	1.06	2.72	46.22	0.00	0.00	0.00	0.00	0.00	1.43	2.71E-08
PPE978	-0.75	15.67	8.57	0.00	2.44	72.13	0.00	0.00	0.00	0.00	0.00	1.18	2.52E-08
PPE979	-0.54	18.78	17.45	2.42	6.34	52.65	0.00	0.00	0.00	0.00	0.00	2.36	3.57E-08
PPE980	-0.43	10.71	12.60	0.86	5.92	69.34	0.00	0.00	0.00	0.00	0.00	0.57	1.96E-08
PPE981	-0.36	22.45	20.67	0.92	4.34	50.29	0.00	0.00	0.00	0.00	0.00	1.33	3.83E-08
PPE982	-0.26	5.34	4.67	1.98	5.87	81.13	0.00	0.00	0.00	0.00	0.00	1.01	1.76E-08
PPE983	-0.2	19.43	15.69	2.85	6.41	54.23	0.00	0.00	0.00	0.00	0.00	1.38	3.46E-08
PPE984	0.05	11.86	5.61	0.63	2.91	76.34	0.41	0.00	0.00	0.00	0.00	2.23	1.76E-08
PP1	0.15	18.66	6.79	4.15	1.44	66.89	0.00	0.00	0.00	0.00	0.00	2.07	5.31E-08
PP2	0.25	20.67	18.02	1.81	6.72	49.19	0.00	0.51	0.00	1.17	0.00	1.90	4.72E-08
PP3	0.4	36.38	22.03	2.35	6.17	30.54	0.00	0.20	0.87	0.00	0.00	1.46	7.60E-08
PP4	0.55	27.39	16.44	9.37	8.75	34.87	0.00	0.17	0.00	0.63	0.00	2.37	6.08E-08
PP5	0.65	29.72	15.88	1.36	4.97	42.92	0.00	0.57	0.00	0.00	0.00	4.57	5.51E-08
PP6	0.75	23.72	20.83	2.98	5.42	43.02	1.78	0.54	0.00	0.00	0.00	1.71	3.89E-08
PP7	0.85	21.76	19.27	1.81	5.45	46.99	1.63	0.00	0.00	0.00	0.00	3.09	4.12E-08
PP8	1.15	19.46	8.90	0.18	3.19	63.22	1.06	0.00	0.00	0.00	0.00	3.99	4.32E-08
PP9	1.3	26.50	17.90	2.47	4.61	45.33	0.00	0.33	0.00	0.00	0.00	2.87	5.14E-08
PP10	1.45	23.12	19.22	2.55	8.30	44.34	0.00	0.00	0.00	0.00	0.00	2.47	4.77E-08
PP11	1.55	29.33	14.33	2.61	9.16	41.33	0.00	0.43	0.00	0.00	0.00	2.81	5.41E-08
PP12	1.95	17.53	13.16	1.95	5.02	60.66	0.00	0.00	0.50	0.00	0.00	1.18	3.85E-08
PP13INF	2.2	33.22	20.33	2.60	4.19	34.89	0.00	0.64	0.00	1.01	0.00	3.11	6.81E-08
PP13MID	2.4	27.90	17.67	1.70	4.36	44.34	0.00	0.54	0.00	1.02	0.00	2.47	6.13E-08
PP13SUP	2.6	31.93	20.23	2.59	5.43	35.22	0.00	0.50	0.00	1.24	0.00	2.85	6.16E-08
PP14	2.95	18.76	15.03	2.56	4.33	57.85	0.00	0.00	0.00	0.00	0.00	1.47	3.11E-08
PP15INF	3.3	21.56	13.67	1.48	3.55	58.33	0.00	0.00	0.00	0.00	0.00	1.41	3.93E-08
PP15MID	3.6	31.56	14.66	0.73	4.21	46.04	0.00	0.57	0.00	0.00	0.00	2.23	4.43E-08
PP15SUP	3.9	29.98	17.34	1.82	4.36	43.03	0.00	0.43	0.00	0.00	0.00	3.05	6.09E-08
PP16	4.35	18.04	11.45	0.61	4.56	62.44	0.00	0.51	0.75	0.00	0.00	1.64	3.76E-08
PP16.20	4.55	33.67	12.00	1.86	1.99	45.17	0.00	0.63	0.00	1.01	0.00	3.66	5.17E-08
PP16.40	4.75	24.66	14.78	6.39	9.92	41.56	0.00	0.62	0.00	0.88	0.00	1.19	5.21E-08
PP16.60	4.95	23.67	17.44	0.47	2.56	53.67	0.00	0.00	0.00	0.00	0.00	2.19	4.76E-08
PP16.80	5.15	33.24	15.23	1.96	5.47	37.90	1.29	0.51	0.60	0.00	0.00	3.80	6.29E-08
PP16.100	5.35	29.12	17.09	1.43	5.76	40.12	4.02	0.00	0.00	0.00	0.00	2.46	5.58E-08
PP16.120	5.55	33.67	13.78	1.92	5.78	38.54	0.00	0.60	0.00	1.51	0.63	3.58	6.14E-08
PP16.140	5.75	32.55	22.10	1.46	5.23	33.24	0.80	0.00	0.00	1.29	0.00	3.34	5.42E-08
PP16.160	5.95	25.99	23.24	2.08	7.86	34.31	0.00	0.52	0.57	2.85	0.00	2.56	5.72E-08
PP16.180	6.15	28.66	13.46	1.87	5.56	41.29	3.70	0.62	0.00	1.79	0.00	3.06	5.96E-08
PP16.200	6.35	22.74	21.45	1.27	3.13	45.78	0.00	0.00	0.00	1.16	1.10	3.37	5.54E-08
PP16.220	6.55	28.43	10.78	0.94	3.23	50.74	2.01	0.00	0.00	1.80	0.00	2.07	5.42E-08
PP16.240	6.75	22.12	9.78	0.70	2.57	59.02	0.00	0.00	0.37	1.41	0.00	4.02	4.58E-08
PP16.260	6.95	20.03	10.67	0.87	4.83	61.66	0.00	0.41	0.00	1.02	0.00	0.51	4.20E-08
PP16.280	7.15	25.20	14.67	1.53	7.98	46.90	0.00	0.00	0.00	1.76	0.00	1.96	4.92E-08
PP16.300	7.35	25.93	13.22	0.62	2.71	55.02	0.00	0.00	0.67	0.00	0.00	1.82	4.68E-08
PP16.320	7.55	33.90	13.78	1.58	5.91	39.02	0.94	0.81	0.00	1.39	0.00	2.66	6.40E-08
PP16.340	7.75	25.05	16.20	1.93	5.07	48.40	0.00	0.00	1.25	0.00	0.95	1.15	5.32E-08
PP16.360	7.95	26.38	19.40	1.17	4.97	43.23	0.94	0.00	0.00	1.34	0.44	2.11	5.98E-08
PP16.380	8.15	24.95	18.19	0.53	4.82	47.02	0.00	0.72	0.59	1.01	0.00	2.16	6.38E-08
PP16.400	8.35	30.78	12.00	3.85	7.64	40.09	1.56	0.98	0.00	0.00	0.69	2.41	6.79E-08
PP16.420	8.55	30.07	19.81	1.74	8.18	35.93	1.22	0.13	0.00	0.95	0.00	1.97	6.33E-08
PP16.440	8.75	29.83	15.77	1.94	3.68	43.17	1.31	0.63	0.00	0.99	0.64	2.04	5.69E-08
PP16.460	8.95	25.70	14.12	1.79	6.58	41.54	1.89	1.01	0.47	2.50	0.00	4.40	5.26E-08
PP16.480	9.15	26.89	27.97	1.94	5.10	32.72	0.00	0.84	0.39	1.24	0.00	2.91	6.25E-08
PP16.500	9.35	27.10	24.29	2.78	7.02	34.89	0.94	0.39	0.00	0.90	0.43	1.26	6.28E-08
PP16.520	9.55	40.21	22.56	1.74	3.64	25.90	0.84	0.70	0.43	0.72	0.00	3.26	8.30E-08
PP16.540	9.75	35.43	16.50	2.78	5.01	33.13	2.43	0.30	1.72	0.77	0.00	1.93	7.43E-08
PP16.560	9.95	30.23	18.19	2.89	6.11	32.40	1.59	0.00	0.00	0.00	0.64	7.95	6.51E-08
PP16.580	10.15	25.03	14.89	1.90	6.54	45.02	1.88	0.86	0.85	1.02	0.00	2.01	5.90E-08
PP16.600	10.35	24.03	13.00	2.09	3.73	48.77	1.09	0.57	0.82	2.59	0.64	2.66	6.24E-08
PP16.620	10.55	32.82	15.10	1.85	8.68	38.67	0.99	0.42	0.00	0.00	0.55	0.92	7.01E-08
PP16.640	10.75												
PP16.660_PT11.30	10.95	52.67	23.78	3.34	6.41	8.10	1.10	0.00	0.00	0.00	0.83	3.78	1.41E-07
PT11.50	11.5	43.67	23.83	3.93	10.78	14.78	2.25	0.00	0.00	0.00	0.00	0.76	1.02E-07
PT11.70	11.7	38.77	27.86	3.02	9.01	16.83	2.30	0.00	0.00	0.00	0.00	2.21	9.48E-08
PT11.90	11.9	36.87	26.48	1.77	7.33	22.15	2.75	0.00	0.00	0.00	1.28	1.38	9.77E-08
PT12.10	12.1	45.22	15.38	1.48	4.62	26.99	1.88	0.00	0.00	1.03	0.69	2.69	9.78E-08
PT12.30	12.3	35.64	18.26	1.29	5.25	34.28	1.59	0.00	0.00	0.00	0.63	3.06	9.25E-08
PT12.50	12.5	52.14	23.14	5.83	5.44	6.43	3.68	0.00	0.00	0.00	1.69	1.65	1.88E-07
PT12.70	12.7	50.89	22.56	3.08	8.29	9.08	3.83	0.00	0.00	0.00	1.33	0.94	1.16E-07
PT12.90	12.9	50.01	22.78	2.68	10.12	9.17	1.64	0.00	0.00	0.89	0.97	1.74	9.57E-08
PT12.90 turb		14.65	32.93	1.39	9.06	36.12	0.71	2.44	0.00	1.18	0.00	1.54	
PT13.10	13.1	49.23	22.56	1.91	5.56	14.89	0.98	0.00	0.00	1.10	1.05	2.73	1.10E-07
PT13.30	13.3	47.89	21.86	3.23	9.17	11.56	2.55	0.00	0.00	0.00	1.00	2.74	1.08E-07
PT13.30 turb		15.73	27.36	4.14	16.20	34.87	0.36	0.00	0.00	0.00	0.23	1.12	
PPT13.45	13.45	44.83	24.93	3.22	5.91	9.63	2.02	0.00	0.00	0.00	0.18	9.29	1.11E-07
PT13.50 turb	13.5	15.18	45.08	26.07	6.30	6.04	0.25	0.00	0.00	0.00	0.24	0.83	5.29E-08
PT13.70 turb	13.7	26.56	40.65	3.94	14.59	9.63	0.90	0.69	0.00	0.00	0.35	2.68	8.53E-08
PT13.90 turb	13.9	28.69	31.89	12.03	11.57	8.62	4.11	0.00	0.00	1.72	0.00	1.36	9.02E-08
PT14.10	14.1	39.52	19.28	2.56	4.51	28.72	2.61	0.00	0.00	0.00	0.00	2.81	9.44E-08
PT14.30	14.3	35.12	16.45	2.07	8.78	31.57	2.22	0.00	0.00	1.21	0.83	1.75	8.56E-08
PT14.50	14.5	27.36	19.23	3.06	10.69	33.82	2.21	0.00	0.00	0.00	0.84	2.80	9.37E-08
PT14.70	14.7	43.33	17.16	2.32	10.58	21.12	3.18	0.00	0.00	0.00	0.92	1.39	1.14E-07
PT14.90	14.9	35.42	20.99	2.45	23.76	12.97	1.22	0.00	0.00	1.70	0.76	0.73	1.15E-07
PT15.10	15.1	19.00	7.55	0.61	3.64	43.74	1.26		0.00	7.12	0.62	0.70	1.13E-07
PT15.30	15.3	44.38	24.17	4.45	4.56	17.96	2.86	0.00	0.00	0.00	0.00	1.62	1.13E-07
PT15.50	15.50	42.34	26.64	2.51	5.64	17.25	2.60	0.00	0.00	0.00	1.02	2.00	1.90E-07
PT15.70	15.70	45.23	20.14	1.96	10.85	16.47	2.08	0.00	0.00	0.00	0.61	2.66	1.42E-07
PT15.90	15.9	50.08	24.55	2.73	8.65	11.12	0.53	0.00	0.00	0.00	1.09	1.26	1.14E-07
PT16.10	16.1	41.45	27.01	3.30	6.54	15.64	2.97	0.85	0.00	0.00	0.00	2.25	8.43E-08
PT16.30	16.3	38.00	21.84	2.21	14.89	16.89	2.50	0.00	0.00	0.00	1.07	2.61	1.14E-07
PT16.50 top turb	16.5	37.35	26.32	3.04	7.59	21.78	2.32	0.00	0.00	0.00	0.00	1.60	1.05E-07
PT16.70	16.7	39.92	21.42	2.39	5.64	22.28	1.95	0.00	3.01	0.00	0.96	2.43	1.00E-07
PT16.90	16.9	41.03	23.46	4.24	7.52	18.56	2.59	0.00	0.00	0.00	0.37	2.24	1.13E-07
PT17.10	17.1	38.47	22.56	2.78	8.86	20.23	2.53	0.00	0.00	1.29	1.03	2.26	1.00E-07
PT17.30	17.3	35.78	30.87	4.34	6.75	16.78	2.23	0.00	0.00	1.13	0.81	1.32	9.23E-08
PT17.50	17.5	25.24	11.64	0.00	2.68	58.00	0.67	0.00	0.00	0.00	0.00	1.76	6.58E-08
PT17.70	17.7	44.06	21.22	3.09	7.56	18.90	2.32	0.00	0.00	0.00	0.83	2.02	1.06E-07
PT17.90	17.9	43.26	21.32	1.95	5.41	25.56	0.74	0.00	0.00	0.00	0.52	1.25	1.06E-07
PT18.10	18.1	45.30	21.91	2.42	6.31	20.01	1.71	0.00	0.00	0.00	0.80	1.54	1.06E-07
PT18.30	18.3	43.56	20.77	4.76	5.42	21.36	1.86	0.00	0.00	0.00	0.39	1.88	1.01E-07
PT18.50	18.5	36.61	18.95	2.15	4.45	33.45	1.08	0.00	0.00	0.00	0.63	2.67	8.99E-08
PT18.70	18.7	36.88	18.26	1.78	5.62	28.82	1.33	0.00	0.00	0.00	0.50	6.81	9.35E-08
PT18.90	18.9	29.41	18.42	3.67	18.92	25.06	2.01	0.77	0.00	0.00	0.76	0.99	8.47E-08
PT19.10 turb	19.1	35.45	25.78	3.45	10.89	15.87	4.02	0.00	0.00	0.80	1.66	2.07	8.39E-08

PT19.10 turb		10.11	16.58	4.09	32.13	28.56	5.01	0.00	0.49	0.00	1.92	1.10	
PT19.20		24.12	20.55	2.53	10.23	31.08	1.64	6.29	0.00	2.23	0.23	1.09	
PT19.30	19.3	40.88	25.44	2.22	10.41	12.47	2.31	0.70	0.00	2.62	0.88	2.07	1.13E-07
PT19.50	19.5	45.26	22.45	4.82	7.29	15.28	1.40	0.88	0.00	0.00	0.94	1.67	1.02E-07
PT19.75	19.7	32.16	30.08	2.04	10.07	20.58	1.05	1.34	0.00	1.16	0.54	0.97	8.96E-08
PT19.90	19.9	32.17	27.89	2.06	5.64	26.87	2.76	0.00	0.00	0.00	0.76	1.85	9.10E-08
PT20.10	20.1	37.53	22.59	3.32	6.12	21.90	1.62	0.00	0.00	0.00	0.75	6.17	8.64E-08
PT20.30	20.3	39.54	26.18	1.81	8.55	19.78	1.07	0.00	0.00	0.00	0.75	2.32	7.85E-08
PT20.50	20.5	31.45	13.30	2.07	3.81	38.98	2.60	2.50	0.00	1.43	1.08	2.78	7.52E-08
PT20.70	20.7	35.44	25.78	5.80	9.87	20.07	1.18	0.00	0.00	0.00	0.37	1.49	8.98E-08
PT20.90	20.9	39.85	21.00	2.07	5.75	25.49	1.24	0.00	0.00	1.15	0.94	2.50	9.01E-08
PT21.10	21.1	29.77	23.87	1.34	13.86	26.45	1.07	0.00	0.00	0.00	1.12	2.52	8.14E-08
PT21.20		34.13	23.77	1.33	9.18	24.84	1.64	1.44	0.00	1.02	0.66	2.00	
PT21.30	21.3	33.58	21.80	2.80	7.59	30.47	1.12	0.00	0.00	0.00	0.80	1.84	8.39E-08
PT21.55	21.5	39.32	21.19	2.30	8.95	23.53	1.29	0.91	0.00	0.00	0.49	2.02	7.14E-08
PT21.60	21.6	24.25	12.46	0.64	2.26	52.17	0.00	1.60	0.00	4.50	0.62	1.50	5.83E-08
PT21.70	21.7	31.31	16.02	1.40	12.78	36.32	0.72	0.00	0.00	0.00	1.34	0.11	7.82E-08
PT21.90	21.9	35.32	23.59	1.69	11.48	25.99	0.00	0.00	0.00	0.00	1.66	0.26	7.92E-08
PT22.00 turb		6.08	39.40	7.59	9.54	35.02	0.00	1.23	0.00	0.00	0.00	1.14	
PT22.10	22.1	28.13	32.49	3.21	7.56	24.72	1.61	0.85	0.00	0.00	0.65	0.77	7.39E-08
PT22.30	22.3	38.92	22.14	2.56	4.25	28.09	2.10	0.00	0.00	0.00	0.80	1.14	8.98E-08
PT22.50	22.5	35.89	33.02	3.21	5.50	19.78	0.60	0.00	0.00	0.00	0.63	1.37	9.10E-08
PT22.70	22.7	27.28	13.36	0.88	3.79	50.23	0.40	0.00	0.00	1.17	0.51	2.39	5.26E-08
PT22.90	22.9	31.10	15.40	1.36	8.24	38.12	1.52	1.69	0.00	1.37	0.31	0.89	5.85E-08
PT22.95		31.05	16.00	2.77	4.91	28.03	2.12	4.18	0.00	9.32	0.56	1.06	
PT23.10	23.1	33.98	22.79	1.59	5.58	32.56	1.29	0.00	0.00	0.00	0.50	1.72	8.09E-08
PT23.30	23.3	36.89	24.03	1.38	5.77	29.78	0.40	0.00	0.00	0.00	0.43	1.32	8.17E-08
PT23.50	23.5	31.20	15.97	1.20	4.77	42.76	1.88	0.00	0.00	0.00	0.00	2.22	6.38E-08
PT23.70	23.7	40.51	19.70	2.93	5.78	27.08	2.56	0.00	0.00	0.00	0.47	0.97	9.57E-08
PT23.90	23.9	38.85	23.47	3.04	5.11	26.47	0.20	0.00	0.00	0.00	0.65	2.20	8.85E-08
PT24.10	24.1	40.12	17.05	2.38	5.42	32.08	1.08	0.00	0.00	0.00	0.45	1.42	7.99E-08
PT24.30	24.3	31.89	18.97	1.60	2.32	41.25	1.07	0.00	0.00	0.00	0.47	2.42	6.84E-08
PT24.50	24.5	36.87	21.00	2.12	4.56	31.09	1.01	0.00	0.00	1.21	0.38	1.76	8.67E-08
PT24.70	24.7	31.34	22.56	3.18	5.54	33.89	1.33	0.00	0.00	0.00	0.00	2.15	8.02E-08
PT24.90	24.9	23.21	14.52	0.83	4.73	52.08	1.72	0.00	0.00	0.00	0.68	2.23	6.37E-08
PT25.10	25.1	32.89	17.78	1.58	9.30	35.55	0.47	0.00	0.00	0.00	0.40	2.02	6.92E-08
PT25.30	25.3	28.36	18.33	1.60	5.58	41.85	1.26	0.00	0.00	0.00	0.50	2.53	6.60E-08
PT25.50	25.5	32.56	19.56	1.99	8.84	33.21	1.17	0.00	0.00	0.00	0.39	2.28	6.57E-08
PT25.70	25.7	31.97	23.97	1.29	4.85	33.98	0.84	0.00	0.00	0.00	0.40	2.70	7.33E-08
PT25.90	25.9	23.76	20.46	1.09	7.01	44.57	0.58	0.00	0.00	0.00	0.35	2.18	5.98E-08
PT26.10	26.1	27.76	14.43	1.25	6.05	47.23	0.60	0.00	0.00	0.00	0.36	2.32	6.12E-08
PT26.30	26.3	32.56	21.89	1.29	18.86	22.78	0.71	0.00	0.00	0.00	0.00	1.91	9.84E-08
PT26.50	26.5	29.61	25.53	0.83	13.03	28.41	1.09	0.00	0.00	0.00	0.46	1.04	8.48E-08
PT26.70	26.7	27.92	20.78	1.33	7.93	39.87	0.37	0.00	0.00	0.00	0.30	1.50	7.29E-08
PT26.90	26.9	20.34	15.89	1.11	2.64	33.23	1.63		0.00	1.79	0.34	12.05	7.97E-08
PT27.10	27.1	37.90	28.98	2.40	8.53	20.12	0.00	0.00	0.00	0.00	0.00	2.07	1.02E-07
PT27.30	27.3	34.50	22.41	1.84	8.89	30.23	0.34	0.00	0.00	0.00	0.57	1.22	8.47E-08
PT27.50	27.5	20.12	7.23	0.91	6.80	61.54	0.80	0.00	0.00	0.00	0.20	2.40	5.55E-08
PT27.70	27.7	27.04	14.09	1.60	2.93	47.76	1.06	0.38	1.06	0.00	0.35	3.73	7.21E-08
PT27.90	27.9	28.90	16.31	1.68	4.87	42.34	1.02	0.44	0.52	1.07	0.00	2.87	6.70E-08
PT28.10	28.1	27.81	17.46	1.83	5.37	41.56	0.86	0.33	1.22	1.26	0.00	2.29	6.82E-08
PT28.30	28.3	29.17	24.32	1.42	8.54	32.56	0.00	0.50	0.00	0.00	0.55	2.93	8.24E-08
PT28.50	28.5	24.86	28.53	12.54	1.52	27.24	0.98	0.40	0.86	0.86	0.43	1.77	8.05E-08
PT28.70	28.7	27.03	18.03	1.35	7.32	40.16	1.21	0.34	0.87	0.00	0.38	3.31	7.00E-08
PT28.90	28.9	29.17	25.97	1.44	7.18	31.47	0.00	0.00	0.00	0.94	0.58	3.26	7.61E-08
PT29.10	29.1	14.56	11.29	17.37	2.40	50.02	1.45	0.00	0.39	0.00	0.00	2.51	4.71E-08
PPT29.3	29.3	34.98	29.55	2.07	4.78	19.90	2.84	0.81	0.83	0.71	0.00	3.52	1.08E-07
PPT29.50	29.5	33.23	25.02	1.18	4.45	29.02	1.05	0.00	1.85	0.74	0.00	3.47	8.82E-08
PPT29.70	29.7	17.02	38.02	2.54	17.02	19.29	0.70	0.27	1.43	1.95	0.28	1.48	5.78E-08
PPT29.90	29.9	31.54	23.54	2.63	10.72	25.02	1.52	0.00	0.31	0.98	0.61	3.13	7.56E-08
PPT30.10	30.1	28.02	22.44	1.90	7.92	33.56	0.00	0.00	0.00	1.11	0.32	4.74	8.44E-08
PPT30.30	30.3	37.12	20.45	1.85	5.34	27.32	2.49	0.19	0.50	0.82	0.00	3.92	9.54E-08
PPT30.50	30.5	30.02	26.67	1.46	10.56	26.12	0.73	0.17	0.59	0.70	0.00	2.99	8.96E-08
PPT30.70	30.7	29.28	26.58	1.91	8.90	27.10	1.04	0.67	0.86	1.50	0.00	2.15	7.90E-08
PPT30.90	30.9	19.11	36.66	1.66	10.90	26.74	0.82	0.00	0.00	0.86	0.41	2.85	6.72E-08
PPT31.10	31.1	21.54	18.04	2.14	26.23	27.28	0.88	0.28	0.53	0.00	0.00	3.09	6.69E-08
PPT31.30	31.3	28.97	20.03	1.34	8.55	34.19	1.06	0.00	0.88	1.49	0.00	3.49	8.18E-08
PPT31.50	31.5	23.90	16.93	2.25	4.96	45.12	0.00	0.28	0.57	1.95	0.60	3.44	7.05E-08
PPT31.70	31.7	23.90	28.45	1.60	15.03	24.66	1.45	0.36	0.72	0.99	0.00	2.84	7.93E-08
PPT31.90	31.9	37.77	25.37	3.54	7.09	20.03	1.64	0.00	0.53	0.00	0.00	4.02	9.43E-08
PPT32.10	32.1	38.97	22.04	3.04	8.34	21.02	0.00	0.38	0.92	0.00	1.60	3.69	8.97E-08
PPT32.30	32.3	34.82	21.33	6.45	6.01	22.22	2.99	0.00	0.98	0.77	0.00	4.43	1.03E-07
PPT32.50	32.5	29.17	24.58	2.22	5.94	30.94	2.08	0.46	0.99	0.87	0.91	1.86	8.27E-08
PPT32.70	32.7	25.04	23.04	1.78	15.03	30.02	1.10	0.00	0.00	0.94	0.41	2.63	7.00E-08
PPT32.90	32.9	25.95	26.75	1.72	4.49	34.02	2.17	0.39	0.69	1.09	0.00	2.73	6.41E-08
PPT33.10	33.1	28.93	22.44	1.97	3.81	36.24	1.04	0.44	0.95	0.76	0.00	3.42	6.89E-08
PPT33.30	33.3	22.03	18.65	3.30	8.00	42.44	0.89	0.41	0.00	1.18	0.00	3.11	6.92E-08
PPT33.50	33.5	27.03	24.88	2.66	7.19	33.12	0.00	0.22	0.57	1.27	0.00	3.06	6.09E-08
PPT33.70	33.7	28.49	25.34	3.01	8.12	31.63	0.00	0.25	0.00	1.43	0.00	1.73	6.75E-08
PPT33.80 turb	33.8	28.14	28.90	1.41	13.56	21.69	0.00	0.53	1.64	1.23	0.00	2.90	6.31E-08
PPT34.10	34.1	36.95	22.08	2.63	3.91	29.18	1.51	0.52	0.58	0.00	0.00	2.65	7.53E-08
PPT34.30	34.3	23.65	12.85	2.05	18.02	37.87	1.10	0.26	0.74	0.00	0.47	3.00	6.75E-08
PPT34.50	34.5	25.03	29.55	1.04	7.90	30.23	0.00	0.50	0.74	0.82	0.27	3.92	7.95E-08
PPT34.70	34.7	24.72	33.10	1.78	11.25	24.04	0.94	0.27	0.00	1.54	0.00	2.37	6.77E-08
PPT34.90	34.9	25.82	21.33	3.22	5.86	40.98	0.79	0.00	0.00	0.00	0.34	1.66	5.96E-08
PPT35.10	35.1	24.90	20.87	1.24	16.04	32.67	0.00	0.33	0.00	1.05	0.00	2.89	7.23E-08
PPT35.30	35.3	37.20	23.67	2.56	5.54	26.89	0.78	0.00	0.00	0.84	0.52	1.99	9.29E-08
PPT35.50	35.5	26.09	18.03	2.77	6.42	42.82	1.02	0.00	0.00	0.00	0.58	2.27	7.44E-08
PPT35.70	35.7	31.49	16.78	1.98	10.37	35.87	0.46	0.00	0.00	0.00	0.28	2.77	7.77E-08
PPT35.90	35.9	30.89	24.77	1.46	7.19	31.83	1.25	0.00	0.00	0.00	0.39	2.22	8.21E-08
PPT36.10	36.1	28.45	32.34	1.87	9.14	25.41	0.86	0.00	0.00	0.00	0.57	1.36	9.02E-08
PPT36.30	36.3	26.67	29.34	2.08	6.22	30.56	1.40	0.00	0.00	1.58	0.00	2.15	8.11E-08
PPT36.50	36.5	22.77	18.60	1.05	4.57	48.76	1.16	0.00	0.00	0.00	0.39	2.70	5.48E-08
PPT36.70	36.7	22.56	29.67	1.38	12.54	30.56	0.43	0.00	0.00	1.83	0.11	0.92	6.18E-08
PPT36.90	36.9	30.43	22.45	3.05	10.45	28.31	0.77	0.00	0.00	1.67	0.69	2.87	6.96E-08
PPT37.10	37.1	20.45	17.22	4.93	8.95	46.33	0.99	0.00	0.00	0.00	0.00	1.13	6.62E-08

This study													
MC1_0	0.00	33.41	23.88	3.35	14.89	16.71	2.76		0.00	2.70	1.08	1.21	8.14E-08
MC1_5	5.00	21.50	29.03	2.40	12.45	26.58	0.83		0.00	5.50	0.12	1.59	6.63E-08
MC1_11	11.00	39.34	26.56	2.56	12.68	13.22	0.65		1.80	1.37	0.00	1.82	9.27E-08
MC1_16	16.00	40.54	31.56	2.62	6.96	11.67	3.24		0.00	1.25	0.59	1.56	1.03E-07
MC1_19	19.00	41.56	31.56	1.87	9.45	9.23	2.25		1.48	1.13	0.95	0.52	9.83E-08
MC1_23	23.00	44.23	31.34	2.25	7.34	10.12	1.37		1.12	0.95	0.70	0.58	1.11E-07
MC1_25	25.00	46.58	24.38	4.12	6.31	12.12	2.10		1.31	0.00	0.84	2.25	1.04E-07
MC1_27	27.00	51.23	22.98	1.74	6.33	13.19	0.57		2.05	0.00	0.70	1.22	1.32E-07
MC1_29	29.00	45.74	25.92	1.57	13.47	10.09	1.65		0.00	0.00	0.50	1.05	1.34E-07
MC1_33	33.00	69.50	2.94	2.69	4.81	10.61	3.65		2.52	0.00	1.29	1.97	1.55E-07
MC1_42	42.00	45.12	28.09	4.08	6.34	10.51	1.96		0.00	1.69	1.06	1.15	9.17E-08
MC1_44	44.00	52.28	22.55	2.82	8.23	10.81	1.18		0.00	0.00	0.85	1.27	9.42E-08
MC1_53	53.00	45.38	32.10	2.25	5.89	9.32	1.50		0.00	0.00	1.42	2.14	8.27E-08
MC2_0	0.00	51.45	26.45	2.68	6.23	7.59	2.98		0.00	1.05	0.41	1.16	1.00E-07
MC2_5	5.00	33.46	33.63	9.44	9.00	9.21	1.67		0.00	1.50	0.69	1.40	8.10E-08
MC2_10	10.00	48.00	22.37	3.38	8.37	8.98	3.11		1.11	1.26	1.33	2.10	9.41E-08
MC2_14	14.00	50.30	24.66	2.56	10.21	8.44	0.70		0.00	0.00	0.84	2.30	1.02E-07
MC2_18	18.00	52.88	21.55	3.26	7.12	9.32	1.43		1.88	0.00	0.84	1.71	1.15E-07
MC2_20	20.00	53.11	28.32	2.54	5.76	7.32	1.16		0.00	0.00	0.99	0.81	1.29E-07
MC2_21	21.00	46.93	29.87	2.07	6.81	10.07	1.38		0.00	0.00	0.86	2.02	1.36E-07
MC2_23	23.00	47.94	32.06	2.15	5.43	7.23	1.17		0.00	0.86	0.88	2.30	1.40E-07
MC2_25	25.00	53.12	26.55	2.15	4.98	9.99	1.20		0.00	0.00	1.16	0.85	1.71E-07
MC2_27	27.00	55.06	25.01	2.71	5.03	6.29	1.34		1.59	0.00	2.04	0.94	1.77E-07
MC2_31	31.00	58.60	25.93	2.27	4.43	5.82	1.30		0.00	0.00	1.26	0.39	1.85E-07
MC2_35	35.00	51.09	28.12	2.66	9.02	4.23	1.94		0.00	0.98	0.49	1.47	1.42E-07
MC2_39	39.00	58.87	20.21	2.79	7.12	8.09	1.68		0.00	0.00	0.29	0.95	1.37E-07
MC2_41	41.00	54.53	21.78	1.62	5.70	13.23	1.64		0.00	0.00	0.80	0.70	1.19E-07
MC2_46	46.00	36.34	23.56	1.85	4.09	31.23	1.40		0.00	0.00	0.64	0.89	8.34E-08
MC2_50	50.00	40.56	19.23	2.01	3.43	32.00	0.97		0.00	0.00	0.28	1.52	8.70E-08
MC2_55	55.00	41.00	13.13	1.69	6.89	31.23	2.11		1.63	0.00	0.89	1.43	8.31E-08
MC2_58	58.00	39.56	18.49	3.31	6.35	26.78	1.52		0.00	1.11	0.71	2.18	9.55E-08
MC3-0	0.00	48.41	23.90	1.62	12.45	10.98	0.00		0.00	0.00	1.01	1.62	1.02E-07
MC3-15	15.00	45.67	22.55	10.28	2.70	13.66	0.00		2.43	0.00	1.31	1.39	1.11E-07
MC3-25	25.00	50.87	20.56	2.97	7.22	12.45	2.01		1.62	0.00	0.93	1.38	1.54E-07
MC3-30	30.00	52.83	20.47	2.61	7.59	9.05	2.97		0.96	0.00	1.31	2.21	1.80E-07
MC3-35	35.00	48.73	25.39	2.84	7.30	11.07	1.17		0.00	0.00	1.00	2.50	1.90E-07
MC3-40	40.00	47.23	19.78	3.28	15.02	8.33	2.10		1.71	0.00	0.96	1.59	1.89E-07
MC3-41	41.00	53.23	22.67	2.41	5.98	8.45	2.15		1.73	0.00	1.07	2.31	1.39E-07
MC3-50	50.00	50.34	22.90	2.13	4.98	13.67	2.73		0.00	0.00	0.97	2.28	1.08E-07
MC3-70	70.00	43.12	20.56	3.67	12.56	14.89	2.09		0.00	0.00	0.98	2.14	1.01E-07

Table S5. Mineralogical data obtained by X-ray diffraction of PT samples (data are from Fantasia et al.[8]f) and of MC samples (this study). Correlation between phyllosilicate and calcite content with χ is shown in Figure S8.

Reference

1 Kruiver, P. P., Dekkers, M. J. & Heslop, D. Quantification of magnetic coercivity components by the analysis of acquisition curves of isothermal remanent magnetisation. *Earth Planet Sc Lett* **189**, 269-276, doi:10.1016/s0012-821x(01)00367-3 (2001).

2 Egli, R. VARIFORC: An optimized protocol for calculating non-regular first-order reversal curve (FORC) diagrams. *Global Planet Change* **110**, 302-320, doi:DOI 10.1016/j.gloplacha.2013.08.003 (2013).

3 Egli, R. Magnetic characterization of geologic materials with first-order reversal-curves. *In Franco, V., Dodrill, B. (Eds.), Magnetic Measurement Techniques for Materials Characterization, in press. Springer Nature Publishing Group.* (2021).

4 Egli, R., Chen, A. P., Winklhofer, M., Kodama, K. P. & Horng, C. S. Detection of noninteracting single domain particles using first-order reversal curve diagrams. *Geochemistry Geophysics Geosystems* **11**, Doi 10.1029/2009gc002916 (2010).

5 Egli, R. Characterization of individual rock magnetic components by analysis of remanence curves, 1. Unmixing natural sediments. *Stud Geophys Geod* **48**, 391-446, Doi. 10.1023/B:Sgeg.0000020839.45304.6d (2004).

6 Egli, R. Analysis of the field dependence of remanent magnetization curves. *Journal of Geophysical Research-Solid Earth* **108**, Doi.10.1029/2002jb002023 (2003).

7 Maxbauer, D. P., Feinberg, J. M. & Fox, D. L. MAX UnMix: A web application for unmixing magnetic coercivity distributions. *Comput Geosci-Uk* **95**, 140-145, doi:10.1016/j.cageo.2016.07.009 (2016).

8 Fantasia, A. *et al.* Global versus local processes during the Pliensbachian-Toarcian transition at the Peniche GSSP, Portugal: A multi-proxy record. *Earth-Science Reviews* **198** (2019).
